# Targeting Cell Senescence and Senolytics: Novel Interventions for Age-Related Endocrine Dysfunction

**DOI:** 10.1210/endrev/bnae010

**Published:** 2024-03-19

**Authors:** Masayoshi Suda, Karl H Paul, Utkarsh Tripathi, Tohru Minamino, Tamara Tchkonia, James L Kirkland

**Affiliations:** Departments of Medicine and Physiology and Biomedical Engineering, Mayo Clinic, Rochester, MN 55905, USA; Department of Cardiovascular Biology and Medicine, Juntendo University Graduate School of Medicine, Tokyo 113-8421, Japan; Departments of Medicine and Physiology and Biomedical Engineering, Mayo Clinic, Rochester, MN 55905, USA; Department of Physiology and Pharmacology, Karolinska Institutet, Solnavägen 9, 171 65 Solna, Sweden; Departments of Medicine and Physiology and Biomedical Engineering, Mayo Clinic, Rochester, MN 55905, USA; Department of Cardiovascular Biology and Medicine, Juntendo University Graduate School of Medicine, Tokyo 113-8421, Japan; Japan Agency for Medical Research and Development-Core Research for Evolutionary Medical Science and Technology (AMED-CREST), Japan Agency for Medical Research and Development, Tokyo, 100-0004, Japan; Departments of Medicine and Physiology and Biomedical Engineering, Mayo Clinic, Rochester, MN 55905, USA; Departments of Medicine and Physiology and Biomedical Engineering, Mayo Clinic, Rochester, MN 55905, USA; Division of General Internal Medicine, Department of Medicine, Mayo Clinic, Rochester, MN 55905, USA

**Keywords:** cellular senescence, endocrine diseases, diabetes, osteoporosis, SASP, senomorphics, senolytics

## Abstract

Multiple changes occur in hormonal regulation with aging and across various endocrine organs. These changes are associated with multiple age-related disorders and diseases. A better understanding of responsible underling biological mechanisms could help in the management of multiple endocrine disorders over and above hormone replacement therapy (HRT). Cellular senescence is involved in multiple biological aging processes and pathologies common in elderly individuals. Cellular senescence, which occurs in many older individuals but also across the lifespan in association with tissue damage, acute and chronic diseases, certain drugs, and genetic syndromes, may contribute to such endocrine disorders as osteoporosis, metabolic syndrome, and type 2 diabetes mellitus. Drugs that selectively induce senescent cell removal, “senolytics,”, and drugs that attenuate the tissue-destructive secretory state of certain senescent cells, “senomorphics,” appear to delay the onset of or alleviate multiple diseases, including but not limited to endocrine disorders such as diabetes, complications of obesity, age-related osteoporosis, and cancers as well as atherosclerosis, chronic kidney disease, neurodegenerative disorders, and many others. More than 30 clinical trials of senolytic and senomorphic agents have already been completed, are underway, or are planned for a variety of indications. Targeting senescent cells is a novel strategy that is distinct from conventional therapies such as HRT, and thus might address unmet medical needs and can potentially amplify effects of established endocrine drug regimens, perhaps allowing for dose decreases and reducing side effects.

## Graphical abstract

**Graphical abstract:**
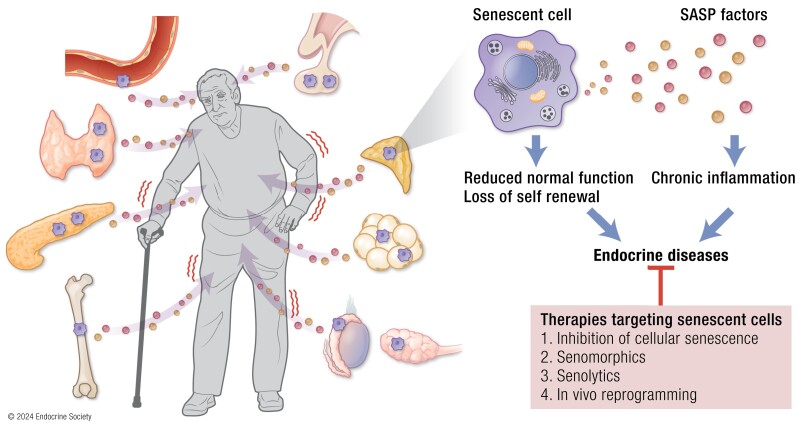

**Essential Points**

- Endocrine organs accumulate senescent cells with increasing age, causing functional decline
- Cellular senescence is a crucial antitumor mechanism, but senescent cell accumulation is detrimental to tissue function
- Senescent cells produce proinflammatory, profibrotic signals dubbed the “senescence associated secretory phenotype”
- Senescent cells can be selectively removed by so-called senolytic drugs, silenced by senomorphic drugs, or reprogrammed back to a nonsenescent state
- Initial results from senolytic clinical trials are promising, but these treatments are new and further studies are warranted, not the least to investigate for potential long-term side effects
- In vivo models targeting senescent cells have been shown to alleviate several endocrine disorders, including diabetes and osteoporosis
- Antisenescence therapies may be an effective adjuvant to existing treatments for endocrine disorders, including but not limited to diabetes mellitus, osteoporosis, infertility, adrenal gland dysfunction, and hypothyroidism

Countries across the world are experiencing growth both in numbers and the proportion of older individuals. By 2050, the world's population of people aged 60 years and older will double (to 2.1 billion) and those aged 80 years or older are expected to triple (to 426 million) (1). Endocrine disorders such as obesity, diabetes mellitus, osteoporosis, and gonadal and thyroid dysfunction are major causes of morbidity and mortality and contribute to an increasingly large health care burden, especially in older individuals (2). In addition, an aging endocrine system contributes to age-associated diseases and geriatric syndromes (3). Associations between increasing age and endocrine function are manifested by declines of several blood hormone levels, such as estrogens (in women), testosterone (in men) (4-6), growth hormone (GH) (7-10), aldosterone (11), and melatonin (12, 13), changes that may correlate with the development of diseases and geriatric syndromes in older patients. For example, low postmenopausal estrogen levels are associated with osteoporosis as well as cardiovascular and cerebrovascular diseases (14, 15), insufficient GH secretion or effects may contribute to loss of skeletal muscle and increased adiposity, decreases in circulating aldosterone to lightheadedness and orthostatic hypotension, and reduced melatonin production to disrupted sleep-wake cycles (ie, the circadian rhythm). On the other hand, the levels of some hormones, such as cortisol (16-18) and thyroid hormones (THs) (19-22), exhibit little negative or positive correlation with increased age. Even when hormone levels are normal or increased, the endocrine system may not function effectively due to changes in target organ function and hormonal responsiveness. For example, insulin resistance and hyperinsulinemia are often present in older individuals (23). Hormone replacement therapy (HRT) has been used to counteract some of these age-related changes in hormone levels or sensitivity (24), although use of HRT can have both benefits and risks (25, 26). How increasing age affects circulating hormone levels requires further study, as new research may disrupt previously held dogmas, indicating the difficulty of drawing generalized conclusions based on studies in younger populations regarding hormone levels in older individuals (20). Recent findings indicate that the association between androgens and aging is not clear. For example, after controlling for comorbidities (27) and aldosterone secretion may increase slightly with aging (28). The complex regulatory systems involved in hormone secretion and activity are influenced by such factors as genetic background, race, ethnicity, sex, lifestyle choices, metabolic factors, and concurrent medical conditions (20). A better understanding about the other biological changes that occur in the endocrine system with aging beyond hormone secretion and processing might help in developing interventions to extend health span, the period of life free of substantial disabilities, chronic disorders and diseases, and decreased physical reserve and resilience.

The effects of cellular senescence and senescent cell accumulation on biological aging processes are becoming increasingly better understood. There is in vitro and in vivo evidence suggesting senescent cell accumulation can contribute to endocrine dysfunction and endocrine disorders such as in the pancreas in type 2 diabetes mellitus (T2DM) (29), adipose tissue in diabetes, obesity, and advanced age (30-37), the kidneys in diabetic nephropathy and obesity (38, 39), the liver in association with hyperinsulinemia, metabolic syndrome, and age-dependent steatosis (40-42), age-related and radiation-induced osteoporosis (43-46), endometrial and uterine dysfunction (47), cancers and their complications, as well as side effects of cancer treatments on endocrine function (48, 49), cardiovascular diseases, including those linked to high-fat diets (50-52), and anxiety associated with obesity (53), among other conditions.

The term “*cellular senescence*” originates from the 1960s, when Hayflick and Moorehead demonstrated that human cultured fibroblasts have limited replicative potential (54). After exhausting their replicative capacity, senescent cells remain metabolically active, and can persist in tissues, causing dysfunction. Apart from not contributing to tissue growth and dampening tissue regeneration due to their cell cycle arrest, senescent cells can release proinflammatory, profibrotic, and proapoptotic signals as well as progrowth factors and factors that modulate progenitor function as part of the senescence-associated secretory phenotype (SASP) (55-58). The proteins, peptides, bioactive small molecules, and noncoding nucleotides that can comprise the SASP are highly variable: The composition of secreted signals depends on the type of cell that became senescent, the trigger that induced senescence, the microenvironment, and time since induction of senescence (59-64).

Targeting and removing senescent cells or reducing the release of certain SASP factors in age-associated diseases have the potential to yield new options for treating diseases without any established effective interventions to date. These novel agents might also be eventually used as adjuvants to HRTs or other therapies, allowing for dose reductions, potentially reducing side effects. Targeting fundamental aging mechanisms might delay, prevent, alleviate, or treat multiple disorders and diseases and complex conditions in older patients including frailty, immobility, sarcopenia/muscle wasting, urinary incontinence, falls, mild cognitive impairment, increased risk for delirium, chronic skin ulcers, loss of resilience against stressors such as surgery and infections, or diminished responses to immunogenic stimuli such as vaccinations (65-69). Since these adverse geriatric conditions are encountered frequently in individuals with diabetes, osteoporosis, and other endocrine conditions (70-72), root-cause treatments targeting fundamental aging mechanisms might be particularly beneficial for such patients.

## Markers of Cellular Senescence

**Figure 1.**
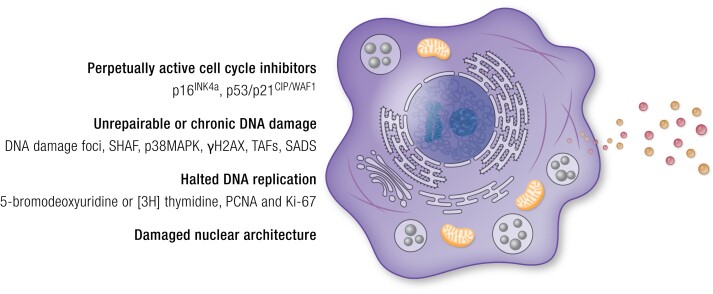
Senescent cell markers. Several senescence markers have been identified based on the molecular biology of senescent cells. Relatively new markers used in recent years are senescence-associated heterochromatin foci, telomere-associated foci, and senescence-associated distention of satellites. However, many of these senescence markers are nonspecific due to the heterogeneity of senescent cells, and to date, no single marker is a fully sensitive and specific indicator, especially for clinical use. Improvements have been made by developing composite scores or signatures comprising multiple markers. Refinement of indicators of senescent cell burden in vivo for clinical application, especially those that can be assayed noninvasively, is needed.

Senescent cells have an enlarged and flattened morphology, frequently have high p53/p21^CIP1/WAF1^ and/or p16^INK4a^/Rb protein levels, and have DNA damage foci (particularly in telomeres) (Fig. 1). Another biomarker for detecting senescent cells in culture or tissue samples is the lysosomal enzyme, senescence-associated-β-galactosidase (SA-βgal), with enzymic activity at pH 6.0 as opposed to lysosomal enzymes from nonsenescent cells (73). However, this marker is not highly sensitive or specific. A central feature of senescence is replicative arrest and lack of DNA replication. This can be detected using clonal plate dilution assays or by the absence of nucleoside analogue incorporation (eg, 5-bromodeoxyuridine or [3H] thymidine) (74). Immunostaining for proliferation markers, such as proliferating cell nuclear antigen and the marker of proliferation, Ki-67 (Ki-67), can also help in detecting senescent cells. Increased proinflammatory SASP factors, including interleukin-6 (IL-6), interleukin-1α (IL-1α), interleukin-8 (IL-8), monocyte chemoattractant protein 1, plasminogen-activated inhibitor 1 (PAI-1), plasminogen-activated inhibitor 2 (PAI-2), and matrix metalloproteinases (MMPs) can indicate increased senescent cell burden and can be analyzed in blood, tissues, or cells (64). However, not all senescent cells are proinflammatory and proapoptotic (63, 75). Hence, there are many different senescence markers with varying degrees of specificity and sensitivity. As of today, there is no single marker for accurately measuring senescent cell accumulation, and the establishment of new cellular senescence markers or composite scores comprising key markers is a pressing issue. Ideally, these markers or scores should be detectible in noninvasively collected samples such as blood and urine.

Senescent cells generally have persistent DNA damage. Nuclear senescence-associated heterochromatin foci can be used to identify senescence induced by activated oncogenes such as H-RAS and BRAF and stressors that impede DNA replication (76, 77). Increased activity of p38 mitogen-activated protein kinase (p38MAPK) or the γ phosphorylated form of the histone H2AX (γH2AX) reflects activated DNA damage responses, which along with depleted or irreparably damaged telomeres, can indicate cellular senescence. DNA damage response factors that persist at sites of damage and nuclear foci, which can be detected cytologically, can also serve as indicators of cellular senescence. Telomere-associated DNA damage foci (TAFs) that accumulate within telomeric sequences, as well as colocalization of γH2AX and p53-binding protein 1 (53BP1) with telomeres, are also indicators of the senescent state. Pericentromeric satellite heterochromatin undergoes decondensation in senescent cells, leading to senescence-associated distention of satellites (SADs). SADs appear earlier and more consistently than heterochromatin foci, reflecting an early and potentially key event in cellular senescence (76, 78). Damage-associated molecular pattern factors, such as high-mobility group box 1 localization or molecules released by stressed cells undergoing cell death, such as mitochondrial DNA (mtDNA), reflect cellular damage and can indicate increased senescent cell abundance (79).

The enzyme α-Klotho regulates multiple endocrine processes, such as insulin-like growth factor 1 (IGF-1) signaling (80), while also modulating mammalian target of rapamycin (mTOR) (81, 82), cyclic adenosine monophosphate (83), p53/p21^CIP1/WAF1^ (84), and Wnt protein levels (85). It is also involved in mineral metabolism, contributing to phosphate homeostasis. α-Klotho has come to light as a geroprotective factor that protects against physiological stresses such as oxidative damage and hypoxia. α-Klotho is also protective against the side effects of cytotoxic drugs. It is secreted by the distal renal tubule and so is present in urine. Importantly, α-Klotho is inversely and causally linked to senescent cell burden, and senolytics increase urinary α-Klotho in humans (86). This suggests that α-Klotho, along with other measures, could be a useful “gerodiagnostic” marker with respect to senescent cell abundance and other fundamental aging processes.

Analysis of microRNAs (miRNAs) that may be specific to senescent cells (87, 88), other nucleotides such as cell-free mtDNA (79), senescence-specific epigenetic profiles, and senescence-associated small extracellular vesicles (EVs) including exosomes (89, 90), microsomes, or mitosomes could also be viable strategies for assessing senescent cell burden. Progress has been made with miRNAs, short (20-24 nt) noncoding RNAs that are involved in posttranscriptional regulation of gene expression. Several miRNAs that are differentially expressed with aging and by senescent cells have been reported (87, 91, 92).

Since none of these markers can be used as a reliable senescence biomarker on its own, combinations of such indicators may be a more reliable reflection of senescent cell burden (74, 93, 94). Such composite scores could be useful for reflecting senescent cell abundance and following therapeutic efficacy in clinical trials of agents targeting senescent cells (95, 96). Cell cycle arrest proteins (eg, p53, p21^CIP1/WAF1^ and p16^INK4a^) can be measured in body fluids. Among these, p16^INK4a^ expression in peripheral blood T cells has been used to estimate senescent cell burden (97, 98). Biopsy samples of, for example, adipose tissue or skin could be an alternative for assessing senescent cell burden, potentially along with imaging modalities.

## Senescent Cells Can Accumulate With Age and Drive Morbidity

**Figure 2.**
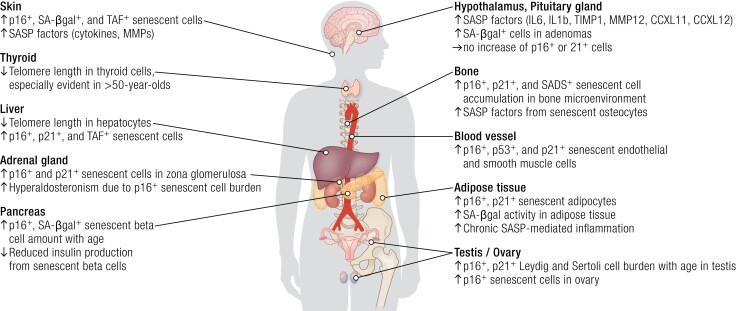
Senescent cell accumulation in endocrine tissues with aging. With increasing age, senescent cells can accumulate in tissues, as demonstrated by assays of *p16^INK4a^/p21^WAF1/CIP1^* expression, increased senescence-associated β-galactosidase activity, or shortened telomeres. Another approach is to monitor levels of senescence-associated secretory phenotype factors. Senescent cells can disrupt tissue homeostasis in endocrine and other organs and dysregulate hormone production and target organ effects, contributing to worsening health outcomes.

Accumulating evidence from in vitro and in vivo studies using the senescent markers mentioned earlier indicate that senescent cells accumulate in multiple endocrine organs with aging, especially in older individuals with impaired function, decreased physical resilience, and/or multimorbidity. Senescent cell accumulation may contribute to the onset and progression of several endocrine diseases (Fig. 2).

### Diabetes Mellitus, Metabolic Syndrome, and Senescence

The prevalence of T2DM increases with age (99, 100), together with the hallmarks of this condition such as increased insulin levels and peripheral insulin resistance. Adipose tissue is a metabolically dynamic organ that is the primary site of excess energy storage, but it also serves as an endocrine organ capable of synthesizing a number of biologically active compounds that regulate metabolic homeostasis, such as tumor necrosis factor-α (TNFα), IL-6, IL-8, leptin, adiponectin, angiotensin, resistin, and PAI-1 (101-103). Two decades ago, it was found that aging is linked to increased adipose tissue inflammation and decreased capacity of cloned adipose progenitors and mesenchymal stem cells for differentiation and replication. This decline in adipogenesis correlated with insulin resistance (104). These findings suggest that presenescent and senescent cells accumulate in adipose tissue with aging since impaired replication is a hallmark of cellular senescence. It was then found that insulin resistance correlates with increased markers of cellular senescence in fat tissue, including adipose tissue β-galactosidase, which is a senescence-linked marker of increased lysosomal activity, as well as increased levels of PAI-1, p53, and cyclin D kinase inhibitors, including p16^Ink4a^ (37, 105, 106). Excessive caloric intake leading to insulin resistance increases SA-βgal activity with increased p53 and p21^CIP1/WAF^ expression in adipose tissue compared to normal caloric intake (105). These changes were related to accumulation of reactive oxygen species (ROS), which can drive senescence or be a product of senescent cells (105). In adipose tissue–specific p53 knockout (KO) mice, insulin resistance caused by a high-fat diet was significantly attenuated, and senescence markers in adipose tissue were decreased even during increased caloric intake (105). These findings suggest that insulin resistance linked to obesity can be mediated by cellular senescence in adipose tissue. Complications of insulin resistance, including hepatic steatosis progressing to nonalcoholic fatty liver disease (NAFLD) and nonalcoholic sclerosing hepatitis (NASH), diabetic/high-fat diet–induced kidney disease, diabetic cardiovascular dysfunction, macular degeneration, and obesity-related neuropsychiatric dysfunction are associated with senescent cell accumulation in the affected organs (34, 38-42, 52, 107, 108).

### Insulin/Insulin-like Growth Factor-1 Signaling and Aging

Examples of hormones affected by aging include insulin and IGF-1, which regulate metabolic balance, anabolic activity, and replication and differentiation of multiple types of cells (109). In 1997, it was reported that a mutation leading to decreased expression of *daf-2* (Dauer formation-2), which encodes an insulin/IGF-1 receptor in the nematode *Caenorhabditis elegans*, results in a 2- to 3-fold increase in lifespan (110, 111). This gene is conserved across species ranging from yeast to mammals (112). Other genes involved in insulin/IGF-1 signaling have also been shown to extend lifespan when suppressed. Examples include the transcription factor, *daf-16*, a *C elegans* homologue of the forkhead box O (*FOXO*) gene in humans that is downstream in the IGF-1/Akt signaling pathway (113, 114), and *Sch9*, a gene homologous to protein kinase B (*Akt*) (115, 116). In *Drosophila*, mutations leading to decreased expression of the insulin/IGF-1 receptor or in insulin receptor substrate (IRS) 2-like molecules have also been shown to be associated with prolonged lifespan (117). Increases in lifespan from reduced insulin signaling are conserved in more complex organisms. In mammals, in which insulin and IGF-1 have separate receptors, heterozygous IGF-1 KO mice live an average of 30% longer than controls (118) and mice lacking adipose tissue insulin receptors live 18% longer, as do mice lacking brain IRS2 (119, 120). Mice with genetic GH deficiency or humans with isolated GH deficiency are protected from aging phenotypes and show longevity (121). The National Institute on Aging's Primate Aging Research Project found that caloric restriction extends lifespan in primates. Caloric restriction of rhesus macaques over a 20-year period resulted in animals that were biologically “younger,” with shinier hair and less likelihood to develop age-related diseases (122). These monkeys also had low circulating insulin (123, 124). These findings suggest that insulin/IGF-1/Akt signaling might have a role in primate aging (122). In the Baltimore Longitudinal Study of Aging, which began in 1958, approximately 700 older men were followed for 25 years. Low insulin levels were associated with longevity (125), further suggesting that insulin/IGF-1/Akt signaling is involved in the development of age-related dysfunction and diseases (126, 127).

Consistent with our Unitary Theory of Fundamental Aging Mechanisms (128, 129), age-related changes in IGF-1 and senescent cell abundance appear to be interlinked (130). The zinc metalloproteinase plasma protein-A (PAPP-A) increases IGF-1 bioavailability by cleaving insulin-like growth factor binding proteins (IGFBPs), particularly IGFBP-4 (131). IGFBPs bind IGF-1, hindering receptor activation. After cleavage of IGFBP-4 by PAPP-A, IGF is liberated from the binding protein and IGF signaling is initiated in the pericellular environment. Inhibiting PAPP-A activity by gene deletion, and thereby decreasing IGF-1 signaling, appears to extend lifespan by up to approximately 30% in naturally aging mice (132). This was also observed in PAPP-A KO mice on a high-fat diet, and even in already adult mice after PAPP-A gene expression was knocked-down (132-134). In the conditioned medium from senescent (senescence induced by etoposide) adult human primary preadipocytes, PAPP-A proteolytic activity was more than 12-fold higher compared to conditioned medium from nonsenescent cells, indicating that PAPP-A is an SASP component. Proteolytically active PAPP-A was also abundant on the surface of EVs secreted by senescent preadipocytes. These findings link increased IGF-1 activity to cellular senescence (130).

Perhaps paradoxically, despite increases in lifespan related to low IGF-1 levels, low levels of circulating IGF-1 in plasma have also been linked to low muscle mass, a key component of frailty (135), although this has not been found consistently and requires further study. Frailty indices include grip strength, gait speed, or other indirect measurements of muscle mass, strength, and function. IGF-1, along with its systemic anabolic effects, directly enhances muscle protein synthesis, driving hypertrophy (136), as well as increasing the abundance of the satellite cells that surround and support skeletal muscle (137). IGF-1, through Akt, increases mTOR activity, which in turn increases protein synthesis (138). It also decreases FOXO levels and thus protein breakdown (139). In a 1990 study of men older than 60 years with low IGF-1 levels, exogenous GH administration increased bone density and lean muscle mass while decreasing adipose tissue mass (140). However, studies with recombinant human GH and GH secretagogues failed to demonstrate benefits that outweigh risks such as increases in insulin resistance linked to weight gain. Resistance training may decrease frailty and improve health in older patients (141-144) and stimulates the hypothalamic-pituitary GH–IGF-1 axis through stimuli from muscles (145). Thus, relationships among IGF-1, muscle function, frailty, health span, and lifespan are complex.

### Osteoporosis and Senescence

With increases in the number of older people, the prevalence of osteoporosis has been increasing. Reduction in bone quality brings with it reduced quality of life, mainly due to pathological fractures (146, 147). The pathophysiology of osteoporosis is multifactorial, and vitamin D deficiency is a risk factor. With age, the capacity of the skin to synthesize vitamin D decreases (148) and senescent cells accumulate (149), but whether this association is causal remains to be determined.

Detrimental effects of senescent cell accumulation in bone have recently come to light. The senescence biomarkers, p16^INK4a^ and p21^CIP1/WAF1^ were elevated in iliac crest needle biopsies from older postmenopausal compared to younger premenopausal women. Despite the heterogeneous nature of the biopsy samples, including variations in cellular composition and fat abundance, SASP factors were also increased in biopsies from the older participants (78). Accumulation of senescent cells in the bone microenvironment is linked to increased bone resorption by osteoclasts and reduced bone formation by osteoblasts, leading to reduced bone density (150). The senescent osteocytes that accumulate with aging in mice have increased expression of multiple SASP factors compared to young mice, as well as age-associated upregulation of SASP factor production in bone marrow myeloid cells. These findings suggest that senescent osteocytes and their SASP may contribute to age-related bone loss and that their removal may be a therapeutic strategy for age-related (as opposed to postmenopausal) osteoporosis (151).

### Hypothalamus and Pituitary Gland

The hypothalamus and the pituitary gland are key regulatory organs of the endocrine system, and this regulatory function can become disrupted with aging. Age-related loss of hypothalamic regulation might be linked to SASP factors, as hypothalamic pro-opiomelanocortin neurons were increased by rapamycin, a known SASP inhibitor (152). In rodents, the hypothalamus becomes less sensitive to several feedback processes with aging (153, 154). Hypothalamic arcuate nucleus GH-releasing hormone secretion is decreased in older individuals while paraventricular nucleus somatostatin secretion is increased, contributing to decreased GH levels related to reduced frequency and lower amplitude of secretory pulses (155-157). In a recent study, age-related changes were detected in the hypothalamic expression of the SASP factors IL-6, IL-1β, TIMP metallopeptidase inhibitor 1 (Timp1), Mmp12, Cxcl1, and Cxcl2. However, the senescent cell markers p16^Ink4a^ and p21^Cip1/Waf1^ were not increased (64). This needs to be explored further at the single-cell level in the hypothalamus because in some tissues, for example, muscle, it was recently discovered that a very small, difficult to detect compartment of senescent cells contributes to substantial dysfunction (158). Deleterious effects of this small senescent cell fraction on muscle function were dramatically alleviated by senolytics.

The diurnal rhythmic release of melatonin is regulated, in part, by a circadian clock located in the suprachiasmatic nucleus of the hypothalamus. This release can become dysregulated with increasing age, leading to decreased levels of melatonin and poor sleep quality (159). In mice, disruption of circadian clock genes such as *Bmal1* or *Clock* reduced lifespan (160-162) and accelerated development of age-related phenotypes. Interestingly, poor sleep quality may be correlated with increased senescent cell burden, evidenced in humans by increased numbers of circulating senescent T cells (163) and increased *p16^INK4a^* gene expressing peripheral blood mononuclear cells (164). In addition, senescent cells can accumulate in the aorta after induced sleep fragmentation (165). More work needs to be conducted to evaluate the connection between sleep disturbances and senescent cells, perhaps focusing on effects of hypothalamic senescent cell burden.

The pituitary gland is prone to adenoma formation with aging, with an estimated prevalence of clinically silent adenomas in older individuals of up to 20% (166). Increased SA-βgal activity has been found in pituitary adenomas compared with normal tissue, possibly due to oncogene-induced senescence (OIS) (167, 168). In this instance, the appearance of senescent cells may be beneficial, since OIS can slow tumor growth. Perhaps this could be the case for other endocrine tumors, but further research into this is needed.

### Gonadal Dysfunction and Sex Hormone Disorders

With increasing age, the ovary and uterus in females and the testis in males become dysfunctional (169), with decreased sexual function, infertility, sleep, and mood disturbances, and loss of muscle mass (170). Gonadal dysfunction with aging may be related to cellular senescence and mitochondrial dysfunction (171), and senolytic or senomorphic interventions may have the potential to delay menopause and extend the female reproductive window (172). In aged rats, p16^Ink4a^ levels increase in the ovaries and testes (173). In aged compared to young dogs, a 4-fold increase in p21^CIP1/WAF1^ expression in testicular fibroblasts and 8 times more senescent Leydig cells in the testes were found (174). Senescent cells may interfere with the differentiation of endometrial stromal cells into decidual cells, impeding embryonic implantation and placentation, leading to infertility (175). However, a recent study using p16^Ink4a^-KO mice found no improvement of infertility in an alkylating agent-induced primary ovarian insufficiency model on inhibiting p16^Ink4a^-induced senescence (176). More work needs to be performed to determine if the accumulation of senescent cells in gonadal tissues causes dysfunction.

### Adrenal Gland

The adrenals secrete cortisone, aldosterone, and dehydroepiandrosterone (DHEA), among other hormones. The connection between DHEA and longevity has been investigated in several epidemiological studies (177-179). In the previously mentioned Baltimore Longitudinal Study, high DHEA levels were associated with longevity (125). Dysregulated function of the adrenals can have wide-reaching consequences, even affecting brain function and mood. The daily cortisol rhythm is disrupted in patients with dementia, along with decreased DHEA levels. High levels of circulating cortisol may induce apoptosis of hippocampal neurons, while high levels of DHEA protect them (180, 181). Senescent cell accumulation in the adrenals has been suggested to have detrimental effects, with senescent cells in the zona glomerulosa being linked to hyperaldosteronism (182, 183). In a recent paper investigating potassium inwardly rectifying channel subfamily J member 5 (KCNJ5)-mutated aldosterone-producing adenomas, p21-induced cell cycle arrest was correlated with higher aldosterone-to-renin ratios (184). In KCNJ5-mutated and wild-type aldosterone-producing adenomas, compact tumor cells were more likely to be senescent than intratumor clear cells (184). More work is needed to test if and how cellular senescence affects adrenal function and tumor formation. Since DHEA replacement therapy in older women did not lead to major benefits (177, 185), widespread use of DHEA supplementation as a gerotherapeutic intervention is not supported as of today.

### Thyroid and Parathyroid Glands

Studies in human and animal models have suggested an inverse relation between TH levels and longevity. Older men appear to have decreased sensitivity to thyrotropes, possibly related to decreased thyrotropin-releasing hormone 24-hour rhythmicity or increased somatostatin (186, 187). In vivo studies in several long-lived small mammalian species suggest that lower TH levels are associated with extended longevity, such as a recent study in which 4 small mammalian species were studied (188). This indicated there is an inverse relation between thyroxine (T4) levels and species maximum lifespan. Increased mitochondrial activity related to TH may link increased TH to accelerated development of aging phenotypes, perhaps related to increased generation of DNA-damaging ROS. This appears to be associated with the accelerated appearance of aging changes in vitro and in vivo (189). ROS and cellular senescence are causally linked. ROS can induce cells to become senescent, and senescent cells activate macrophage degradation of nicotinamide adenine dinucleotide (NAD), which then leads to increased ROS generation, especially by those innate immune cells that can induce other types of nonsenescent cells to become senescent (190, 191). Studies are needed to determine if these interlinked cellular-senescence–mediated processes are accentuated by TH. Also pointing to potential links between senescence and the effects of thyroid function on the progression of aging phenotypes is the observation that caloric restriction, which can delay the development of aging phenotypes, both reduces circulating TH and decreases senescent cell burden (173). Progressive telomere shortening, which can be both a cause and biomarker of cellular senescence, has been noted in the thyroid and parathyroid glands of humans. Thyroid and parathyroid telomeric erosion becomes evident in people older than 50 years, which is later than for other tissues in which telomere shortening has been reported (192). Perhaps this is due to the slow turnover of thyroid cells. Older women tend to have higher thyrotropin (TSH) levels than younger women and, in both sexes, triiodothyronine (T3) tends to be lower and circulating antithyroid antibodies higher in older than younger populations (193). In geographic areas where iodine intake is high, TSH levels tend to increase with age, whereas in areas with lower iodine intake, circulating TSH levels generally decrease with increasing age (193, 194). Long-term residency in areas with high iodine content in the drinking water has been associated with increased longevity (195-197). Although epidemiological data suggest a relationship between iodine intake and longevity, little is currently known about whether this is an indirect effect of changes in TH or iodine homeostasis.

The most aggressive thyroid neoplasm is anaplastic thyroid cancer (ATC). Historically, the mean survival time after this diagnosis has been established is 4 months (198). In the thyroid gland, type 2 deiodinase (D2) converts T4 into the more metabolically active T3, which is crucial for ATC cell proliferation. Interestingly, treatment by the D2 inhibitor, reverse T3, induces cellular senescence in these tumor cells (184). Together with senolytic therapy to remove these now-senescent cancer cells in a 2-step process (“1:2 punch” approach) (199, 200), this may become a valid treatment strategy for patients suffering from this type of cancer.

### Vitamin D Metabolism

The conversion of the vitamin D precursor, 7-dehydrocholesterol, into previtamin D_3_ occurs in the skin with exposure to ultraviolet radiation in sunlight. The capacity of the skin to synthesize previtamin D_3_ decreases by up to 50% with increased age (148, 201, 202). Cellular senescence plays a major role in skin aging, with senescent cells accumulating that produce collagenases and elastase that disrupt skin architecture and contribute to altered skin pigmentation (203, 204). As such, senescent cell accumulation in the skin may theoretically contribute to reduced vitamin D production, a possibility that needs to be tested. The ability of the kidneys to complete the synthesis of metabolically active vitamin D is also reduced with age (205-208). Vitamin D deficiency is widespread worldwide (209) and has been connected to obesity (210) and osteoporosis (211), among other endocrine disorders. Interestingly, vitamin D has potent geroprotective effects, with higher vitamin D levels correlating with longer telomeres in humans (212). Vitamin D has been shown to reduce senescent cell burden by inhibiting the p16 and p53 pathways (213).

### Thymus

The thymus is effectively an endocrine organ as well as a vital component of the immune system since it produces hormones such as thymulin, thymosin, and thymopoietin, and the thymus is the first organ in the body that exhibits age-associated involution. Studies have shown that cellular senescence occurs in thymic epithelial cells due to high oxidative stress, especially during advanced stages of human thymic involution (214). The senescence indicators, p16^Ink4a^, p53, p21, enhanced SA-βgal activity, and γ-H2AX were detected by immunohistochemistry in the aged human and mouse thymus (214-217). Age-related thymic involution contributes to immunosenescence and inflammaging declines due to the capacity to establish central tolerance, thereby causing increased self-reactive T cells to escape to the periphery (218). Much work remains to be done to test whether there are causal links between cellular senescence and endocrine and immune function of the thymus.

### Other Organs Related to Endocrine Diseases

Vascular endothelial cells (ECs) are abundant throughout the body and produce vasoactive peptide hormones, growth factors, coagulation factors, and adhesion molecules, effectively functioning in an endocrine-like manner. In a recent in vitro study, EC-conditioned media were collected from cultures treated with radiation to induce senescence in nonsenescent ECs. The senescent ECs had a more robust SASP than senescent epithelial cells or myoblasts. Senescent ECs also exhibited functional abnormalities, including decreased expression of endothelial nitric oxide synthase and increased ROS production (219). Production of prostenoids such as prostaglandin I2 was decreased, whereas production of PAI-1, thromboxane-A2, and endothelin-1 (was increased (220-222). Furthermore, atherosclerotic coronary arteries have increased SA-βgal (223) and *p16^INK4a^* and *p53/p21^CIP1/WAF1^* expression (224-228).

Senescent cells accumulate in the placenta during pregnancy (229, 230), and senescence can spread locally and systemically (231). Vascular senescence is prominent in preeclampsia, which is linked to a disrupted fetal-maternal barrier that in turn is linked to metabolic dysfunction, hypertension, recurrence of preeclampsia during subsequent pregnancies, and accelerated development of aging phenotypes in women (232-234). The effect of senescent vascular cells on the action of vasoactive and other hormones requires further study.

## Therapies Targeting Senescent Cells

Cellular senescence has an important role in several conditions related to aging as well as multiple diseases across the lifespan (30, 128, 129, 204, 235). Dietary and exercise interventions can prevent the accumulation of senescent cells by reducing DNA damage, mitochondrial dysfunction, excessive ROS, and inflammation (236) or reduce deleterious properties of senescent cells, such as attenuating the SASP (237). Caloric restriction was one of the first lifespan-extending interventions to be identified. Other dietary interventions including methionine restriction (238) and ketogenic diets (239, 240) promote a favorable metabolic state that may limit the accumulation of senescent cells. Exercise is another promising strategy to delay aging phenotypes with numerous health benefits by preventing the accumulation of senescent cells (241-243). It has been shown that low-magnitude vibration, which mimics exercises, can alleviate age-related bone loss by inhibiting senescence of osteogenic cells in aged rats (244). However, whether these interventions have direct effects on senescent cells requires further study, as these interventions are multifaceted. In addition, since many reports showed that exercise activates intrinsic immune cells (245-247), these improvements on aging phenotypes may be due to the indirect removal of senescent cells by intrinsic immune cell activation (248).

**Figure 3.**
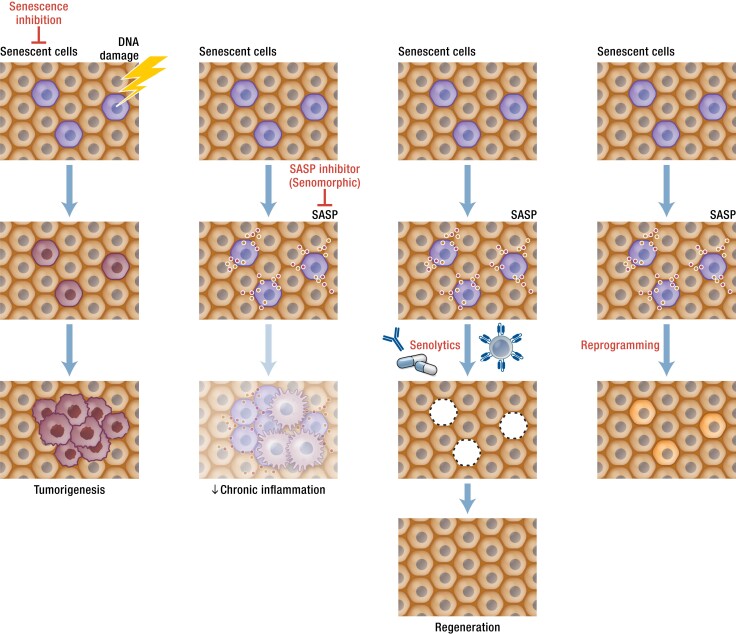
Scheme of senotherapies. Four strategies for attenuating detrimental effects of senescent cells. The first, targeting the senescence program itself, may lead to increased tumor formation due to apoptosis-resistant, damaged, cancerous mutation–harboring cells continuing to proliferate, and as such is not being as widely investigated currently as other approaches. The second strategy is to modify the characteristics of senescent cells using senomorphic agents that decrease production of tissue-damaging senescence-associated secretory phenotype (SASP) factors. This can be achieved using already available agents such as metformin, rapamycin, or ruxolitinib, but as the senescent cells remain in tissues, more continuous administration of senomorphic drugs may be required compared to senolytics. The third option is to target the apoptosis resistance mechanisms operative in the 30% to 70% of senescent cells that are tissue-damaging with senolytic drugs, leading to their removal. Due to the time it takes for new senescent cells to form and acquire a proapoptotic SASP, senolytics are effective even if administered intermittently using a “hit-and-run” approach. The fourth option is to alter the epigenetic programming of senescent cells by induction of Yamanaka Factors (OSKM), reverting them into a nonsenescent, replicating state. This approach, if perfected, could be promising, but since many senescent cells can harbor or develop oncogenic mutations, it could lead to cancers, including teratocarcinomas.

Besides these nonpharmacological approaches, currently 4 approaches for targeting senescent cells are being investigated in preclinical studies (Fig. 3): (1) inhibition of senescent cell formation; (2) suppression of the SASP (senomorphics); (3) elimination of persisting senescent cells (senolytics); and (4) reprogramming of senescent cells.

### Inhibition of Cellular Senescence

Targeting the cell cycle inhibitors that enforce cellular senescence directly, such as p53, which is upstream of p21^CIP1/WAF1^, may prevent cells from becoming senescent. Using p53-conditional KO mice, inhibiting cellular senescence in an adipose and vascular EC-specific manner attenuated obesity and alleviated glucose intolerance (105, 224, 225, 249). However, systemically reducing *p16^Ink4^*^a^ or *p53-p21^Cip1/Waf1^* expression is likely to increase cancer risk since *p53*, *p21^Cip1/Waf1^*, and *p16^Ink4a^* global KO mice have a high prevalence of cancer and hence a shorter lifespan than control mice (250-253). Local or intermittent administration of agents that inhibit senescent cell formation might be an alternative, but even a few DNA-damaged cells with revived proliferative potential might still be sufficient to cause cancer. Hence, interfering with the capacity of cells to become senescent, as opposed to removing already senescent cells (which can include senescent cells harboring cancerous mutations), may not become a viable strategy because of the role of induction of senescence in preventing replication of cancerous cells.

### Suppression of the Senescence-associated Secretory Phenotype (Senomorphics)

As some SASP factors have a role in chronic inflammation and the progression of multiple disorders, SASP inhibitors, also called senomorphic agents, can break this link between proinflammatory, proapoptotic senescent cells and disease without directly eliminating senescent cells. Several senomorphics target the transcription factor nuclear factor (NF)-κB, Janus kinase (JAK), or the JAK signal transducer and activator of transcription (STAT) signaling pathways. Other senomorphics target rapamycin complex 1 (mammalian target of rapamycin complex 1; mTORC1), mitochondrial complex 1- or 4-related (eg, metformin), or p38 mitogen-activated protein kinase (MAPK) family members. Further possibilities for inhibiting the SASP include modulating NAD^+^/NADH metabolism, inhibiting heat shock protein 90, or neutralizing SASP factors or their receptors (254). A recently described *p21^Cip1/Waf1^-Cre* mouse model, which has a *p21^Cip1/Waf1^* promoter driving inducible *Cre*, enables direct targeting of *p21^Cip1/Waf1^*-highly expressing (p21^high^) senescent cells (255). Using this model, it was demonstrated that inactivating the NF-κB pathway in p21^high^ cells attenuated insulin resistance in obese mice, as did the removal of p21^high^ cells, suggesting that SASP factors, over and above senescent cells themselves, may be a central contributor to insulin resistance (256).

Rapamycin is approved as an immunosuppressant at high doses. It or related mTORC1 inhibitors used at much lower, less immune-system–suppressing doses, appear to be senomorphic and decrease frailty (257), heart failure (258), cancer formation (259), cognitive impairment (260), immune dysfunction (261), and age-related adipose tissue loss. Rapamycin also appears to increase the maximum lifespan in mice (at least in animals raised under ideal, constant, nonstressed, pathogen-free conditions that may not reflect the real world) (262). Ruxolitinib is an inhibitor of the JAK1/JAK2-STAT3 pathway, which has been implicated in cellular senescence (263). Ruxolitinib is in clinical use for various disorders (eg, polycythemia rubra vera, myelofibrosis, and graft-versus*-*host disease). Ruxolitinib inhibits production of some SASP factors in vitro and in vivo in aged mice (264). In rodents, it alleviates age-related adipose tissue dysfunction, decreases insulin resistance, reduces age-related osteoporosis and frailty, alleviates critical illness myopathy, and decreases progenitor cell dysfunction (35, 265). In older myeloproliferative syndrome patients, ruxolitinib partially attenuates frailty and increases appetite, body weight, and skeletal muscle strength, although it does not directly affect the hematological disorder itself. This indicates that ruxolitinib may alleviate frailty and geriatric phenotypes through mechanisms such as SASP inhibition independently of its effects on hematological function (266).

Metformin, an inexpensive drug that has been used to treat diabetes for more than 60 years, reduces the release of multiple proinflammatory SASP factors by senescent cells, with NF-κB inhibition playing a key role (267). Metformin has been shown to delay, prevent, or alleviate multiple age-related disorders, including cardiovascular diseases (268), cognitive dysfunction (269), and diabetes in animals and humans (270). A retrospective analysis of patients with diabetes who received metformin suggested there could be an increase in lifespan compared to individuals without diabetes (271). The proposed TAME (Targeting Aging with Metformin) clinical trial will test if metformin delays the appearance of a second age-related disease in patients who already have a single age-related condition (272, 273).

Disentangling the effects due to SASP modulation by these agents from other “off-target” effects that could lead to side effects is difficult. Examples of potential adverse off-target effects of senomorphics may include suppression of cytokine secretion by nonsenescent immune cells (eg, in the case of rapamycin), potentially hindering physiologically necessary inflammation in the face of an infection, or inhibition of anabolism, potentially contributing to reduced myogenesis under some conditions. In addition, since the senescent cells themselves remain in the body, continuous administration of SASP inhibitors may be necessary, potentially leading to more side effects than senolytics, which appear to be effective even if administered intermittently. However, it should be noted that some senomorphics may be effective intermittently (274), perhaps because they decrease the SASP-induced spread of senescence (discussed next).

### Elimination of Senescent Cells (Senolytics)

The idea of selectively targeting and removing senescent cells was suggested by a study that indicated interventions such as caloric restriction that enhance health span or lifespan are associated with decreased senescent cell burden in mice (173). Based on that earlier study and after work to develop senolytic agents that selectively eliminate senescent cells had already begun, this speculation was reinforced by the observation that removing cells highly expressing *p16^Ink4a^*, many or perhaps most of which are senescent cells, enhances function in transgenic mice with an accelerated aging-like state due to transgenic expression of a gene predisposing to DNA mutations (275). In these *INK-ATTAC* mice, administration of a drug that has little or no effect on cells lacking the *ATTAC* transgene (first used in 2005 in *FAT-ATTAC* mice to remove fat cells) (276), highly expressing *p16^Ink4a^* cells undergo apoptosis. This observation in progeroid mice suggested that cellular senescence might be implicated in generating age-related phenotypes in naturally aging mice and that removal of senescent cells might prevent or delay cellular senescence-related dysfunction (277, 278). This was first demonstrated across multiple age-related disorders in naturally aged mice treated with senolytic agents (discussed next) that clear senescent cells (75) and confirmed in naturally aged *INK-ATTAC* mice, in which targeting highly expressing *p16^Ink4a^* cells alleviated senescence-related metabolic dysfunction (35). Fulfilling Koch's postulates of causality, it was shown that transplanting small numbers of senescent cells into middle-aged mice is sufficient to cause frailty, physical dysfunction, and the premature onset of most or all of the diseases that older nontransplanted mice normally die from (231). It was further shown that transplanting senescent cells causes the recipient's own cells to undergo senescence, even at a distance. Hence, senescence can spread not only in a paracrine but also distantly in an “endocrine” fashion. Additionally, intermittently eliminating senescent cells alleviated physical dysfunction after senescent cell transplantation, all pointing toward senescent cells as a causal contributor to aging phenotypes and diseases (231). Possible benefits of selectively eliminating senescent cells were subsequently indicated in mouse models using other markers of aging such as p21^Cip1/Waf1^ (255, 256) and p19 (279). These findings support a potential role for targeting senescent cells as a therapeutic strategy to delay, prevent, alleviate, or treat multiple age-related pathologies, including endocrine and metabolic diseases and disorders (68, 129).

As senescent cells are more apoptosis-resistant than nonsenescent cells (280), it was theorized that they may have increased expression of prosurvival signals, defending them against their own proapoptotic SASP factors (75). To test this, proteomic data from different types of human senescent cells were analyzed and compared to nonsenescent cells. Using bioinformatics approaches, these data were further analyzed with the goal of identifying specific senescent cell antiapoptotic pathways (SCAPs). It was discovered that different SCAPs are upregulated depending on the senescent cell type. Next, key identified SCAP nodes were inhibited using small interfering RNAs (siRNAs). This induced apoptosis in the 30% to 70% of senescent cells that are proapoptotic and tissue-damaging (75). These SCAP components included ephrins (EFNB1 or 3)/SRC kinases, the phosphatidylinositol-4,5-bisphosphate 3-kinase delta catalytic subunit (PI3KCD), the cyclin-dependent kinase inhibitor 1A (CDKN1A; p21^CIP1/WAF1^), BCL-xL, mitochondrial pathways, and plasminogen-activated inhibitor-2 (PAI-2) (75, 281-284). Next, bioinformatics approaches were used to select small molecules that could target the identified SCAPs. Dasatinib (D), a drug approved by the Food and Drug Administration since 2006 for hematologic malignancies, is a kinase inhibitor that interferes with the SRC kinase/EFNB1/3-dependent apoptosis resistance of senescent human fat cell progenitors, leading to their selective apoptosis (285, 286). Quercetin (Q) is a natural flavonoid in apple skin, capers, and red onions that is available in the United States and Europe as a health supplement. Q is known to interfere with PI3K, and this mechanism was shown to induce death in tissue-damaging senescent human ECs (75). The combination of dasatinib and quercetin (D + Q) was theorized to have wider senolytic activity than either alone and this combination successfully reduced senescent cell burden in chronologically aged mice (75). Based on the findings regarding BCL-xL in the aforementioned siRNA studies, 8 months later 2 groups found that navitoclax (ABT-263) also exhibited senolytic activity against certain types of senescent cells and could alleviate age-related disorders and dysfunction (287, 288). To date, multiple additional senolytics have been identified using the original hypothesis-based drug discovery approach and, later, by high-throughput screening. Examples include the specific BCL-xL inhibitors A1331852 and A1155463 (289), the flavonoid fisetin (289, 290), piperlongumine (282), procyanidin C1 (291), and FOXO4-related peptide (292), among others. Several dozen small-molecule senolytics have now been identified (128). Since senescent cells can take from 1 to 6 weeks to fully develop, at least in vitro, and senescent cells do not replicate, senolytics can be administered once every couple of weeks or once a month. In mouse models, this appears to be as or more effective than administering these agents continuously (30, 128, 150).

Most current senolytics act on SCAP pathways. Second-generation senolytics are now being identified using high-throughput library screens and other approaches, including cardiac glycosides, such as digoxin, which was found to decrease the number of senescent cells after senescent cell implantation in mice (293, 294). Other recently identified senolytic targets include glutaminase 1 (295), GPNMB (glycoprotein nonmetastatic melanoma protein B) (226, 296), CD153 (297), uPAR (urokinase-type plasminogen activator receptor) (298), and intravenous zoledronic acid, a bisphosphonate that is both senolytic and senomorphic (299).

### Direct In Vivo Partial Reprogramming

Another strategy targeting aging processes being tested by some research groups is cellular reprogramming using the “Yamanaka factors,” Oct4, Sox2, Klf4, and c-Myc (OSKM) (300). This strategy, which uses these transcription factors to bring senescent cells closer to a stem cell–like state, may convert the senescent cells accumulated in endocrine organs into functional cells. This reprogramming of senescent cells may be a viable treatment for several endocrine diseases, such as diabetes. In a murine model, with mice modified with an OSKM polycystronic cassette and in which OSKM expression can be induced on doxycycline treatment, OSKM induction increased pancreatic β-cell regenerative capacity and reduced metabolic dysfunction caused by streptozotocin. In older wild-type mice, reprogramming enhanced muscle regeneration after cardiotoxin-induced muscular injury (301), indicating that reprogramming may alleviate sarcopenia, much like intermittent oral senolytic D + Q administration (158). The field is still new, and it remains to be seen if it can be made safer and if creating cancers, such as malignant teratomas, by allowing potentially cancer-harboring senescent cells to reenter the cell cycle can be avoided.

## Preclinical Models Targeting Endocrine Senescence

Cellular senescence has been implicated in various endocrine disorders, and preclinical data showing improvement of endocrine and related disorders with senolytics are accumulating (Table 1). Ongoing research in the senescence field may, apart from these common conditions, also possibly yield new treatment options against relatively rarer diseases, such as hypothalamic and pituitary gland disorders and aldosterone-secreting adenomas, along with several other endocrine disorders, but little is currently known about these rarer diseases as there are either no adequate preclinical models or existing models are difficult to evaluate. The evidence summarized here is for senolytics in animal models of more common conditions.

**Table 1.** Results from preclinical studies of senolytics in endocrine diseases

Preclinical models of therapies targeting senescent cells in endocrine organs or endocrine-related diseases			
Endocrine organ or endocrine-related disease	Model name	Treatment	Result
Pancreas	*INK-ATTAC*	Navitoclax	Improved β-cell function
Adipose tissue	*INK-ATTAC* *p16-3MR*	D + Q	Improved metabolic and adipose tissue function, reduced inflammation, improved adipogenesis
Bone	*INK-ATTAC*	D + Q	Higher bone mass and strength and better bone microarchitecture
Frailty	*INK-ATTAC*	D + Q	Improved and delayed age-associated physical dysfunction Improved muscle growth after resistance training in old age
Pituitary gland	*Hesx1^Cre/+^;Ctnnb1^lox(ex3)/+^*	Navitoclax, ABT-737	Reduced adamantinomatous craniopharyngioma explant size after senolytic treatment
Testis	Aged	FOXO4-DRI	Improved testicular microenvironment, alleviated age-related testosterone secretion insufficiency
Ovary	Cisplatin treated	D + Q	Significant improvement of ovarian function

**Abbreviation:** D + Q, dasatinib and quercetin.

### Senolytics for Diabetes Mellitus and Metabolic Syndrome

In diet-induced obese mice, senescent cell clearance has beneficial effects on adipose tissue function and systemic metabolism, with the ratio of subcutaneous to visceral fat being increased without reducing total body weight (30, 34). Senescent cell clearance also decreased lipid deposition in muscle and liver (34, 42). These changes and the extent of senescent cell clearance correlated with enhanced insulin sensitivity (34). Senescent cell clearance in aged mice mitigated age-related subcutaneous fat tissue atrophy by enhancing adipogenesis (35). In addition to these metabolic benefits, senolytics also prevented or alleviated some complications of diabetes in obese mice, including diastolic cardiac dysfunction, hepatic steatosis, microalbuminuria, and obesity-induced anxiety (34, 35, 39, 42). Clinical trials of senolytics for diabetes, adipose dysfunction, and their complications are underway (Table 2).

**Table 2.** Clinical studies of senotherapies for endocrine-related diseases

Clinical studies with therapies targeting senescent cells on endocrine or endocrine-related diseases					
Targeted endocrine or endocrine-related disease	Study title	Senolytic Study	Design	Identifier	Status
Adipose tissue dysfunction	Senescence in chronic kidney diseases	D + Q	Phase 2, randomized, open-label	NCT02848131	Current, preliminary report published
Diabetic kidney disease	Inflammation and stem cells in diabetic and chronic kidney disease	Fisetin	Phase 2, randomized, double-blind, placebo-controlled	NCT03325322	Current
Insulin resistance	Alleviation by Fisetin of Frailty, Inflammation, and Related Measures in Older Women (AFFIRM)	Fisetin	Phase 2, randomized, double-blind, placebo-controlled	NCT03430037 NCT03675724	Current
Diabetic ophthalmopathy	Safety and tolerability study of UBX1325 in patients with diabetic macular edema or neovascular age-related macular degeneration	UBX1325	Phase 1, open-label Phase 2, randomized, double-blind, sham-controlled	NCT04537884 NCT04857996	Current
Osteoporosis/Frailty	Targeting cellular senescence with senolytics to improve skeletal health in older humans and Alleviation by Fisetin of Frailty, Inflammation, and Related Measures in Older Women (AFFIRM)	D + Q; Fisetin	Phase 2, randomized, open-label, Phase 2, randomized, double-blind, placebo-controlled	NCT04313634 NCT03430037 NCT03675724	Current
Epigenetic aging of all tissues	Safety and Effectiveness of Quercetin & Dasatinib on Epigenetic Aging	D + Q	Phase 1, open-label, prospective nonrandomized	NCT04946383	Active, not recruiting
NAFLD	Dasatinib and Quercetin to Treat Fibrotic Non-alcoholic Fatty Liver Disease	D + Q	Phase 1, double-blind, randomized, controlled, proof-of-principle	NCT05506488	Not yet recruiting
Platinum-resistant or refractory ovarian cancer	A Study of ABT-263 as Single Agent in Women With Platinum Resistant/Refractory Recurrent Ovarian Cancer (MONAVI-1)	Navitoclax	Phase 2, interventional single group assessment	NCT02591095	Published
Prostate cancer	Navitoclax and in Treating Patients With Progressive Metastatic Castrate Refractory Prostate Cancer	Navitoclax	Phase 2 open-label parallel assignment	NCT01828476	Terminated
Aging (diabetes, heart disease, Alzheimer, dementia, cancer)	Targeting Aging with Metformin (TAME) Trial	Metformin	Phase 3, randomized, placebo-controlled		Not yet recruiting

Abbreviation: D + Q, dasatinib and quercetin.

### Senolytics and Osteoporosis, Decreased Motor Function

Intermittent senolytic treatment in old mice reduced *p16^Ink4a^* expression and SADS in osteocyte-enriched bone samples (78). Both genetic and pharmacologic removal of senescent cells as well as the SASP inhibitor, ruxolitinib, suppressed cortical bone resorption, increased bone formation on endocortical surfaces, and enabled bone maintenance on trabecular surfaces of some bones. This resulted in improved bone microarchitecture and increased bone strength (44, 150). Senolytics increased osteoblast numbers and bone formation rates while reducing bone marrow adipose tissue. This was due to alterations in progenitor commitment toward osteoblast formation and away from osteoclast and adipocyte formation following senescent cell clearance. Senolytics also alleviated frailty symptoms in these mice (75, 302), indicating far-reaching effects of senescent cell removal, even when targeting a single disease. Clinical trials of senolytics for age-related osteoporosis are underway (see Table 2).

### Senolytics and the Pituitary Gland

In the pituitary tumor, adamantinomatous craniopharyngioma (ACP), cells that carry oncogenic β-catenin mutations and have increased Wnt signaling, form cell clusters that become senescent and promote SASP factor production. Evidence supporting paracrine signaling by senescent cells as a risk factor for tumorigenesis across different tumors and cancer models is accumulating (303). In ex vivo cultures of pretumoral pituitary glands, the senolytic compounds navitoclax and ABT-737 tended to reduce the average size of the clusters (304) and the cardiac glycosides ouabain and digoxin, which also are senolytic compounds, killed oncogenic β-catenin–expressing cells and reduced the levels of senescence markers and SASP factors (293). These data suggest that senolytics may synergize with established anticancer drugs by eliminating OIS cells.

### Senolytics and Fertility

In a small study of endometrial tissue from patients with endometriosis, D alone, Q alone, and especially D + Q reduced senescence markers and enhanced decidualization marker expression, suggesting further studies for infertility are warranted (175).

## Clinical Studies of Senotherapies for Endocrine Disorders

### Clinical Trials of Senolytics

Based on promising results in preclinical experiments in cultured human cells, human tissue explants, and animals, clinical studies are already underway (128). The first senolytic clinical trial published was an open-label pilot study in patients with idiopathic pulmonary fibrosis, conducted because idiopathic pulmonary fibrosis is a progressive and fatal disease with no highly effective treatment. Fourteen patients were treated with intermittent oral D + Q (100 mg/day of D and 1250 mg/day of Q) for a total of 9 doses: 3 days each week for 3 weeks in a brief, non–placebo-controlled, open-label study of safety and tolerability (305). The results suggested that senolytic treatment might alleviate physical dysfunction (gait speed, gait distance, chair stands, and short physical performance battery) in patients with idiopathic pulmonary fibrosis. However, the study was not placebo-controlled and phase 2 studies are needed. Post hoc analysis of another study involving 20 patients with idiopathic pulmonary fibrosis revealed higher levels of the geroprotective factor α-Klotho in urine after oral D + Q administration in each of the 20 participants (86).

Another pilot study involving patients with diabetic kidney disease used, for the first time, a composite score developed for assaying senescent cell burden in humans. Nine individuals with diabetic kidney disease treated with a 3-day course of oral D + Q (100 mg/day of D and 1000 mg/day of Q) had decreased senescent cell burden in fat tissue compared to before the administration of senolytics (95). The fat tissue was biopsied 11 days after the last dose (95). There was also a decrease in circulating SASP factors 11 days after the last dose of D + Q compared to before D + Q was administered. Of note, D has a 3-hour and Q an 11-hour elimination half-life, so the agents were no longer present at the time of the second adipose biopsy and blood collection. This study indicated that the D + Q senolytic combination is effective in reducing senescent cell accumulation and associated inflammation in humans, suggesting that an intermittent, hit-and-run strategy may be a viable approach (95).

After these promising results, more than 30 clinical trials of senolytic therapies for a variety of diseases are planned, ongoing, or completed, including phase 2 randomized, double-blinded, placebo-controlled trials (128). Other senolytics such as fisetin as well as D + Q will be used in upcoming or ongoing phase 2 trials. If positive, these trials will have to be followed by larger clinical trials examining the effects of senolytic drugs on senescence-related disorders and diseases.

### Potential Clinical Use of Senotherapies for Endocrine Diseases

Although results from preclinical data appear promising with respect to certain endocrine and related diseases, there are limited human clinical study data at this point. This is because the senotherapeutic field is new and the clinical trials conducted so far have been limited in scale since they are pilot studies. Examples of clinical trials currently planned or underway for endocrine disorders, metabolic disorders, and related diseases are in Table 2.

Following the positive results from the pilot study, fisetin as well as D + Q will be used in phase 2 trials for diabetic kidney disease. One study will examine the effect of 20 mg/kg/day of fisetin on adipose tissue-derived mesenchymal stem/stromal cell function, kidney function, systemic inflammation, and physical function in individuals with advanced chronic kidney disease. Another study will evaluate whether targeting systemic senescent cell burden by 100 mg D + 1000 mg Q daily helps reduce markers of insulin resistance, inflammation, bone resorption, and physical dysfunction in older women with gait disturbance.

Besides diabetic kidney disease, osteoporosis is a promising target for senolytic interventions based on preclinical findings, and a clinical trial is now underway to examine age-related osteoporosis. In this trial, markers of senescent cell burden alongside bone formation and resorption markers in older women are being measured. D + Q (D 100 mg/day and Q 1000 mg/day) or fisetin (20 mg/kg/day) for 3 consecutive days is being administered to older women. Type I collagen is being assayed as a serum bone metabolism marker after 20 weeks. The Alleviation by Fisetin of Frailty, Inflammation, and Related Measures (AFFIRM) study is currently being conducted to explore effects of senolytics on musculoskeletal aging and frailty. Fisetin (20 mg/kg/day) for 3 consecutive days is being administered, and serum inflammation markers, bone resorption, insulin resistance, and gait speed are being assayed as outcomes.

In the planned “Safety and effectiveness of Quercetin & Dasatinib on epigenetic aging” study, 500 mg Q and 50 mg D for 3 days in a row per month, for a total duration of 6 months, will be administered to healthy individuals and epigenetic age will be assessed using blood samples.

NAFLD, while not a primary endocrine disorder, is a complication of obesity and insulin resistance for which effective therapeutic agents are lacking. Senolytics may be effective, as suggested by analyses in murine models that indicated decreased hepatic steatosis after senolytic treatment (25). Neither NAFLD nor the related NASH have effective treatments that can reverse these disease processes. A planned double-blinded, randomized, proof-of-principle clinical study will examine the effect of D + Q on liver fibrosis in individuals with biopsy-proven NAFLD (Table 2). In addition to senolytics, the TAME (Targeting Aging with Metformin) clinical trial is planned to test if metformin, a senomorphic drug, delays the appearance of a second age-related disease in patients without diabetes. Other clinical trials of senolytics and senomorphics are in Table 2.

Senolytics may also prove to be effective for cancer-associated conditions, potentially including endocrine cancers (199, 200). As mentioned earlier, in some malignancies such as ACP, it is theorized that tumor cells can induce senescence in surrounding cells, which contributes to the malignant effects of the tumor due to SASP factor release (306). However, SASP factors can also contribute to tumor suppression by causing inflammation and recruiting immune cells that remove these damaged or oncogene-expressing cells (307). Senolytic drugs work very similarly to established anticancer treatments, and in some cases such as dasatinib and navitoclax, are repurposed anticancer drugs. There are 2 planned clinical studies using navitoclax as an adjunctive anticancer drug for cisplatin resistant/refractory ovarian cancer and metastatic castration-refractory prostate cancer. Navitoclax monotherapy has been shown to have poor activity against recurrent epithelial ovarian cancer, but there have been no unacceptable side effects. Another trial for prostate cancer was terminated early (NCT01828476). Although further research is needed on this topic and these trials were not conducted with senolytic effects in mind, senolytic therapies may be an effective adjunctive cancer treatment after toxicity and long-term side effects have been examined.

### Future Directions for Interventions Targeting Senescent Cells

The senolytic treatments in many of the aforementioned clinical studies have to date had only mild side effects and off-target effects. To balance risk with potential benefits, so far clinical trials have focused on serious disorders and diseases for which few treatment options exist, rather than preventive studies in healthy individuals. In addition, there are 2 major issues to be solved before getting to the point where the clinical use of senotherapies can be advocated. First, even though some of the early clinical trials have suggested short-term safety and target engagement for senolytics, the field is still new. Larger, randomized clinical trials are needed for detecting any longer-term adverse effects and determining if these agents are effective. In our opinion, the only context for administering these agents is in carefully controlled clinical trials with data monitoring and safety oversight. Senolytics and other senotherapies should not be prescribed or used over the counter. There is a possibility that many of the early studies will fail. These trials should be viewed as a first step and a basis for continuous improvement of trial design so that as better treatments are developed, these trials can be used as a template. Several of the agents being administered in these early studies are approved for other uses in humans or are natural products with robust safety data. In our view, this needs to be the initial approach rather than beginning with new drugs rarely used in humans before. With these cautions, setbacks of the type that slowed clinical translation of gene therapies due to serious side effects in early trials might be avoided.

The second issue is gerodiagnostic tests. To facilitate the development of interventions, further work in refining gerodiagnostic tests measuring senescent cell burden and the extent of other fundamental aging processes is required. Mere “aging clocks” will be less informative than signatures or composite scores of analytes that change in response to interventions, predict and track clinical changes, indicate which intervention to use, are reliable, reproducible, scalable, and inexpensive, and can be assayed in urine, saliva, blood, buccal swabs, or other easily obtained samples. There is not any single, universal, or fully specific biomarker for identifying senescent cells reliably in cell culture or tissue samples because the phenotype of senescent cells varies considerably among cell types and tissues. New approaches are being explored that take the initial cell type into account to subclassify senescent cells and focus on distinct senescent cell types. The initial trigger for senescence may determine the phenotype of senescent cells, an example being adipocyte senescence induced by high-fat diets, in which upregulation of p21^CIP1/WAF1^ but not so much of p16^INK4a^ occurs (255, 256). To characterize senescence fully and develop more sensitive and specific markers of different senescent cell subtypes, transcriptomic and proteomic studies down to the single-cell level across relevant cell and tissue types will be of importance, facilitating delineation of cell surface molecules that will allow detection and isolation of senescent cells from blood or tissue samples. Another solution to this problem is to combine sets of SASP factors and other senescence-associated analytes into a composite score to identify and quantify senescent cells. Several such panels have been developed, the first being one that successfully tracked decreases in senescent cell burden in a clinical trial of senolytics (95). Frequent components of these panels are p21^CIP1/WAF1^ or p16^INK4a^ levels, along with inflammatory SASP factors such as TNFα or IL-1α. Another such composite score is the “SenMayo” panel, which appears to identify senescent cells across different tissues and species (96). This panel can be used at the tissue and single-cell level and identifies key signaling pathways. Panels such as SenMayo may facilitate monitoring senescent cell burden with aging and in diseases and for analyzing the effect of therapies targeting cellular senescence during clinical trials.

## Conclusions

In recent years, research targeting fundamental aging processes has made remarkable progress, and senescent cell-targeting therapies including SASP inhibitors, senolytics, and reprogramming are promising. With accumulating evidence from preclinical studies, some of these therapies have progressed to the point of early clinical trials. Diabetes, metabolic syndrome, macular degeneration, and osteoporosis are among endocrine and endocrine-related disorders for which clinical senolytic trials are planned or ongoing.

Senotherapies are a new treatment strategy and have the potential to significantly change existing treatments. Targeting senescent cells is an intervention against a root-cause contributor to the diseases of aging and may improve age-related conditions in a comprehensive manner, rather than one disease at a time. On the other hand, this field is new, and there are many issues such as long-term outcomes, side effects, and the establishment of gerodiagnostics, so we must proceed carefully. Adoption by the Food and Drug Administration or the World Health Organization International Classification of Diseases code for impaired functional capacity would help in obtaining and tracking data at an epidemiological level and facilitating regulatory oversight. If interventions or combinations or interventions (eg, lifestyle plus drug) can be developed that target interlinked fundamental aging processes additively or synergistically, their first use will likely be for serious diseases and disorders linked to these processes. Eventually, if safe and effective, senotherapies may move toward use for less-serious conditions, as agents coadministered with other interventions already developed to treat particular diseases, and perhaps, eventually, for secondary prevention and even primary prevention to enhance health span.

## Abbreviations

53BP1: Tumor suppressor p53-binding protein 1
γH2AX: γ phosphorylated form of the histone H2AX
ACP: adamantinomatous craniopharyngioma
AFFIRM: Alleviation by Fisetin of Frailty, Inflammation, and Related Measures
Akt: protein kinase B
Ang II: angiotensin II
ApoE: apolipoprotein E
ATC: anaplastic thyroid cancer
ATTAC: apoptosis through targeted activation of caspase 8
BCL-xL: B-cell lymphoma-extra large
BPTES: bis-2-(5-phenyl- acetamido-13,4-thiadiazol-2-yl) ethyl sulfide
CAR-T: chimeric antigen receptor T cell
CDK: cyclin dependent kinase
CDKI: cyclin-dependent kinase inhibitor
CTL: cytotoxic T lymphocyte
Cxcl1: chemokine ligand 1
Cxcl2: chemokine ligand 2
D: dasatinib
D2: deiodinase
DHEA: dehydroepiandrosterone
EC: endothelial cell
EFNB1: ephrin B1
EV: extracellular vesicle
FOXO: forkhead box O
GH: growth hormone
GPNMB: glycoprotein nonmetastatic melanoma protein B
HGPS: Hutchinson-Gilford progeria syndrome
HRT: hormone replacement therapy
IGF-1: insulin-like growth factor 1
IGFBP: insulin-like growth factor-binding protein
IL-α: interleukin-α
IL-6: interleukin-6
IL-8: interleukin-8
IRS2: insulin receptor substrate 2
JAK: Janus kinase
KCNJ5: potassium inwardly rectifying channel subfamily J member 5
KGA: kidney-type glutaminase
Ki-67: marker of proliferation Ki-67
KO: knockout
MAPK: mitogen-activated protein kinase
miRNA: microRNA
MMP: matrix metallopeptidase
mtDNA: mitochondrial DNA
mTOR: mammalian target of rapamycin
mTORC1: mammalian target of rapamycin complex 1
NAD: nicotinamide adenine dinucleotide
NAFLD: nonalcoholic fatty liver disease
NASH: nonalcoholic steatohepatitis
NF: nuclear factor
NK: natural killer
OIS: oncogene-induced senescence
OSKM: Oct4, Sox2, Klf4, and c-Myc
p16^Ink4a^: cyclin-dependent kinase inhibitor 2A
p19^INK4d^: cyclin-dependent kinase 4 inhibitor D
p21^CIP1/WAF1^: cyclin-dependent kinase inhibitor 1 (Cdkn1a)
p38MAPK: p38 mitogen-activated protein kinase
p53: cellular tumor antigen p53
53BP1: p53-binding protein 1
PAI-1: plasminogen activator inhibitor-1
PAI-2: plasminogen activator inhibitor-2
PAPP-A: pregnancy-associated plasma protein A
PI3K: phosphoinositide 3-kinases
PI3KCD: phosphatidylinositol-4,5-bisphosphate 3-kinase delta catalytic subunit
Q: quercetin
Rb: retinoblastoma protein
ROS: reactive oxygen species
SADS: senescence-associated distention of satellites
SASP: senescence-associated secretory phenotype
SA-βgal: senescence-associated β-galactosidase
SCAPs: senescent cell antiapoptotic pathways
siRNA: small interfering RNA
STAT JAK: signal transducer and activator of transcription
T2DM: type 2 diabetes mellitus
T3: triiodothyronine
T4: thyroxine
TAFs: telomere-associated foci
TAME: targeting aging with metformin
TH: thyroid hormone
Timp1: TIMP metallopeptidase inhibitor 1
TIS: therapy-induced senescent cells
TSH: thyrotropin
TNFα: tumor necrosis factor α
uPAR: urokinase-type plasminogen activator receptor

## References

[1] WHO . Ageing and health. Accessed April 5, 2024. https://www.who.int/news-room/fact-sheets/detail/ageing-and-health

[2] Niccoli T, Partridge L. Ageing as a risk factor for disease. Curr Biol. 2012;22(17):R741‐R752. 22975005 10.1016/j.cub.2012.07.024

[3] Cappola AR, Auchus RJ, El-Hajj Fuleihan G, et al Hormones and aging: an endocrine society scientific statement. J Clin Endocrinol Metab. 2023;108(8):1835‐1874. 37326526 10.1210/clinem/dgad225 PMC11491666

[4] Horstman AM, Dillon EL, Urban RJ, Sheffield-Moore M. The role of androgens and estrogens on healthy aging and longevity. J Gerontol A Biol Sci Med Sci. 2012;67(11):1140‐1152. 22451474 10.1093/gerona/gls068 PMC3636678

[5] Orentreich N, Brind JL, Rizer RL, Vogelman JH. Age changes and sex differences in serum dehydroepiandrosterone sulfate concentrations throughout adulthood. J Clin Endocrinol Metab. 1984;59(3):551‐555. 6235241 10.1210/jcem-59-3-551

[6] Baulieu EE . Dehydroepiandrosterone (DHEA): a fountain of youth? J Clin Endocrinol Metab. 1996;81(9):3147‐3151. 8784058 10.1210/jcem.81.9.8784058

[7] Giustina A, Veldhuis JD. Pathophysiology of the neuroregulation of growth hormone secretion in experimental animals and the human. Endocr Rev. 1998;19(6):717‐797. 9861545 10.1210/edrv.19.6.0353

[8] Nass R, Farhy LS, Liu J, et al Age-dependent decline in acyl-ghrelin concentrations and reduced association of acyl-ghrelin and growth hormone in healthy older adults. J Clin Endocrinol Metab. 2014;99(2):602‐608. 24285677 10.1210/jc.2013-3158 PMC3913814

[9] Rudman D, Kutner MH, Rogers CM, Lubin MF, Fleming GA, Bain RP. Impaired growth hormone secretion in the adult population: relation to age and adiposity. J Clin Invest. 1981;67(5):1361‐1369. 7194884 10.1172/JCI110164 PMC370702

[10] Ho KY, Evans WS, Blizzard RM, et al Effects of sex and age on the 24-hour profile of growth hormone secretion in man: importance of endogenous estradiol concentrations. J Clin Endocrinol Metab. 1987;64(1):51‐58. 3782436 10.1210/jcem-64-1-51

[11] Hegstad R, Brown RD, Jiang NS, et al Aging and aldosterone. Am J Med. 1983;74(3):442‐448. 6338717 10.1016/0002-9343(83)90971-3

[12] Karasek M . Melatonin, human aging, and age-related diseases. Exp Gerontol. 2004;39(11-12):1723‐1729. 15582288 10.1016/j.exger.2004.04.012

[13] Martín Giménez VM, de Las Heras N, Lahera V, Tresguerres JAF, Reiter RJ, Manucha W. Melatonin as an anti-aging therapy for age-related cardiovascular and neurodegenerative diseases. Front Aging Neurosci. 2022;14:888292. 35721030 10.3389/fnagi.2022.888292 PMC9204094

[14] Mishra SR, Chung HF, Waller M, Mishra GD. Duration of estrogen exposure during reproductive years, age at menarche and age at menopause, and risk of cardiovascular disease events, all-cause and cardiovascular mortality: a systematic review and meta-analysis. BJOG. 2021;128(5):809‐821. 32965759 10.1111/1471-0528.16524

[15] Lobo RA, Davis SR, De Villiers TJ, et al Prevention of diseases after menopause. Climacteric. 2014;17(5):540‐556. 24969415 10.3109/13697137.2014.933411

[16] Deuschle M, Gotthardt U, Schweiger U, et al With aging in humans the activity of the hypothalamus-pituitary-adrenal system increases and its diurnal amplitude flattens. Life Sci. 1997;61(22):2239‐2246. 9393943 10.1016/s0024-3205(97)00926-0

[17] Veldhuis JD, Sharma A, Roelfsema F. Age-dependent and gender-dependent regulation of hypothalamic-adrenocorticotropic-adrenal axis. Endocrinol Metab Clin North Am. 2013;42(2):201‐225. 23702398 10.1016/j.ecl.2013.02.002 PMC3675779

[18] Purnell JQ, Brandon DD, Isabelle LM, Loriaux DL, Samuels MH. Association of 24-hour cortisol production rates, cortisol-binding globulin, and plasma-free cortisol levels with body composition, leptin levels, and aging in adult men and women. J Clin Endocrinol Metab. 2004;89(1):281‐287. 14715862 10.1210/jc.2003-030440

[19] Waring AC, Arnold AM, Newman AB, Bùzková P, Hirsch C, Cappola AR. Longitudinal changes in thyroid function in the oldest old and survival: the cardiovascular health study all-stars study. J Clin Endocrinol Metab. 2012;97(11):3944‐3950. 22879629 10.1210/jc.2012-2481 PMC3485600

[20] van den Beld AW, Kaufman JM, Zillikens MC, Lamberts SWJ, Egan JM, van der Lely AJ. The physiology of endocrine systems with ageing. Lancet Diabetes Endocrinol. 2018;6(8):647‐658. 30017799 10.1016/S2213-8587(18)30026-3 PMC6089223

[21] Bremner AP, Feddema P, Leedman PJ, et al Age-related changes in thyroid function: a longitudinal study of a community-based cohort. J Clin Endocrinol Metab. 2012;97(5):1554‐1562. 22344200 10.1210/jc.2011-3020

[22] Surks MI, Hollowell JG. Age-specific distribution of serum thyrotropin and antithyroid antibodies in the US population: implications for the prevalence of subclinical hypothyroidism. J Clin Endocrinol Metab. 2007;92(12):4575‐4582. 17911171 10.1210/jc.2007-1499

[23] Al-Sofiani ME, Ganji SS, Kalyani RR. Body composition changes in diabetes and aging. J Diabetes Complications. 2019;33(6):451‐459. 31003924 10.1016/j.jdiacomp.2019.03.007 PMC6690191

[24] Santen RJ, Allred DC, Ardoin SP, et al Postmenopausal hormone therapy: an Endocrine Society scientific statement. J Clin Endocrinol Metab. 2010;95(7 Suppl 1):s1‐s66. 20566620 10.1210/jc.2009-2509 PMC6287288

[25] Basaria S, Coviello AD, Travison TG, et al Adverse events associated with testosterone administration. N Engl J Med. 2010;363(2):109‐122. 20592293 10.1056/NEJMoa1000485 PMC3440621

[26] Liu H, Bravata DM, Olkin I, et al Systematic review: the safety and efficacy of growth hormone in the healthy elderly. Ann Intern Med. 2007;146(2):104‐115. 17227934 10.7326/0003-4819-146-2-200701160-00005

[27] Carto CA, Gurayah AA, Arbelaez MCS, Grewal MR, Kohn T, Ramasamy R. Association between comorbidities and longitudinal changes in total testosterone among men from the Baltimore longitudinal study of aging. J Sex Med. 2023;20(5):605‐611. 36897060 10.1093/jsxmed/qdad025

[28] Nanba K, Vaidya A, Williams GH, Zheng I, Else T, Rainey WE. Age-related autonomous aldosteronism. Circulation. 2017;136(4):347‐355. 28566337 10.1161/CIRCULATIONAHA.117.028201 PMC5568806

[29] Gunasekaran U, Gannon M. Type 2 diabetes and the aging pancreatic beta cell. Aging (Albany NY). 2011;3(6):565‐575. 21765202 10.18632/aging.100350 PMC3164365

[30] Palmer AK, Tchkonia T, Kirkland JL. Targeting cellular senescence in metabolic disease. Mol Metab. 2022;66:101601. 36116755 10.1016/j.molmet.2022.101601 PMC9520013

[31] Wang L, Wang B, Gasek NS, et al Targeting p21(Cip1) highly expressing cells in adipose tissue alleviates insulin resistance in obesity. Cell Metab. 2022;34(1):75‐89.e8. 34813734 10.1016/j.cmet.2021.11.002 PMC8732323

[32] Chaib S, Tchkonia T, Kirkland JL. Obesity, senescence, and senolytics. Handb Exp Pharmacol. 2022;274:165‐180. 34697668 10.1007/164_2021_555

[33] Conley SM, Hickson LJ, Kellogg TA, et al Human obesity induces dysfunction and early senescence in adipose tissue-derived mesenchymal stromal/stem cells. Front Cell Dev Biol. 2020;8:197. 32274385 10.3389/fcell.2020.00197 PMC7113401

[34] Palmer AK, Xu M, Zhu Y, et al Targeting senescent cells alleviates obesity-induced metabolic dysfunction. Aging Cell. 2019;18(3):e12950. 30907060 10.1111/acel.12950 PMC6516193

[35] Xu M, Palmer AK, Ding H, et al Targeting senescent cells enhances adipogenesis and metabolic function in old age. Elife. 2015;4:e12997. 26687007 10.7554/eLife.12997 PMC4758946

[36] Escande C, Nin V, Pirtskhalava T, et al Deleted in breast cancer 1 regulates cellular senescence during obesity. Aging Cell. 2014;13(5):951‐953. 24992635 10.1111/acel.12235 PMC4172532

[37] Tchkonia T, Morbeck DE, Von Zglinicki T, et al Fat tissue, aging, and cellular senescence. Aging Cell. 2010;9(5):667‐684. 20701600 10.1111/j.1474-9726.2010.00608.x PMC2941545

[38] Bian X, Griffin TP, Zhu X, et al Senescence marker activin A is increased in human diabetic kidney disease: association with kidney function and potential implications for therapy. BMJ Open Diabetes Res Care. 2019;7(1):e000720. 10.1136/bmjdrc-2019-000720 PMC6936543 31908790

[39] Kim SR, Jiang K, Ogrodnik M, et al Increased renal cellular senescence in murine high-fat diet: effect of the senolytic drug quercetin. Transl Res. 2019;213:112‐123. 31356770 10.1016/j.trsl.2019.07.005 PMC6783353

[40] Meijnikman AS, van Olden CC, Aydin Ö, et al Hyperinsulinemia is highly associated with markers of hepatocytic senescence in two independent cohorts. Diabetes. 2022;71(9):1929‐1936. 35713877 10.2337/db21-1076 PMC9450852

[41] Baboota RK, Rawshani A, Bonnet L, et al BMP4 and Gremlin 1 regulate hepatic cell senescence during clinical progression of NAFLD/NASH. Nat Metab. 2022;4(8):1007‐1021. 35995996 10.1038/s42255-022-00620-x PMC9398907

[42] Ogrodnik M, Miwa S, Tchkonia T, et al Cellular senescence drives age-dependent hepatic steatosis. Nat Commun. 2017;8(1):15691. 28608850 10.1038/ncomms15691 PMC5474745

[43] Farr JN, Rowsey JL, Eckhardt BA, et al Independent roles of estrogen deficiency and cellular senescence in the pathogenesis of osteoporosis: evidence in young adult mice and older humans. J Bone Miner Res. 2019;34(8):1407‐1418. 30913313 10.1002/jbmr.3729 PMC6697189

[44] Khosla S, Farr JN, Kirkland JL. Inhibiting cellular senescence: a new therapeutic paradigm for age-related osteoporosis. J Clin Endocrinol Metab. 2018;103(4):1282‐1290. 29425296 10.1210/jc.2017-02694 PMC6276719

[45] Chandra A, Lagnado AB, Farr JN, et al Targeted reduction of senescent cell burden alleviates focal radiotherapy-related bone loss. J Bone Miner Res. 2020;35(6):1119‐1131. 32023351 10.1002/jbmr.3978 PMC7357625

[46] Chandra A, Lagnado AB, Farr JN, et al Targeted clearance of p21- but not p16-positive senescent cells prevents radiation-induced osteoporosis and increased marrow adiposity. Aging Cell. 2022;21(5):e13602. 35363946 10.1111/acel.13602 PMC9124310

[47] Cavalcante MB, Saccon TD, Nunes ADC, et al Dasatinib plus quercetin prevents uterine age-related dysfunction and fibrosis in mice. Aging (Albany NY). 2020;12(3):2711‐2722. 31955151 10.18632/aging.102772 PMC7041753

[48] de Magalhães JP . How ageing processes influence cancer. Nat Rev Cancer. 2013;13(5):357‐365. 23612461 10.1038/nrc3497

[49] Cupit-Link MC, Kirkland JL, Ness KK, et al Biology of premature ageing in survivors of cancer. ESMO Open. 2017;2(5):e000250. 29326844 10.1136/esmoopen-2017-000250 PMC5757468

[50] North BJ, Sinclair DA. The intersection between aging and cardiovascular disease. Circ Res. 2012;110(8):1097‐1108. 22499900 10.1161/CIRCRESAHA.111.246876 PMC3366686

[51] Lewis-McDougall FC, Ruchaya PJ, Domenjo-Vila E, et al Aged-senescent cells contribute to impaired heart regeneration. Aging Cell. 2019;18(3):e12931. 30854802 10.1111/acel.12931 PMC6516154

[52] Yu S, Kim SR, Jiang K, et al Quercetin reverses cardiac systolic dysfunction in mice fed with a high-fat diet: role of angiogenesis. Oxid Med Cell Longev. 2021;2021:8875729. 33688395 10.1155/2021/8875729 PMC7914089

[53] Ogrodnik M, Zhu Y, Langhi LGP, et al Obesity-induced cellular senescence drives anxiety and impairs neurogenesis. Cell Metab. 2019;29(5):1061‐1077.e8. 30612898 10.1016/j.cmet.2018.12.008 PMC6509403

[54] Hayflick L, Moorhead PS. The serial cultivation of human diploid cell strains. Exp Cell Res. 1961;25(3):585‐621. 13905658 10.1016/0014-4827(61)90192-6

[55] Campisi J, d’Adda di Fagagna F. Cellular senescence: when bad things happen to good cells. Nat Rev Mol Cell Biol. 2007;8(9):729‐740. 17667954 10.1038/nrm2233

[56] Young AR, Narita M. SASP reflects senescence. EMBO Rep. 2009;10(3):228‐230. 19218920 10.1038/embor.2009.22 PMC2658552

[57] Coppé JP, Patil CK, Rodier F, et al Senescence-associated secretory phenotypes reveal cell-nonautonomous functions of oncogenic RAS and the p53 tumor suppressor. PLoS Biol. 2008;6(12):2853‐2868. 19053174 10.1371/journal.pbio.0060301 PMC2592359

[58] Tchkonia T, Zhu Y, van Deursen J, Campisi J, Kirkland JL. Cellular senescence and the senescent secretory phenotype: therapeutic opportunities. J Clin Invest. 2013;123(3):966‐972. 23454759 10.1172/JCI64098 PMC3582125

[59] Basisty N, Kale A, Jeon OH, et al A proteomic atlas of senescence-associated secretomes for aging biomarker development. PLoS Biol. 2020;18(1):e3000599. 31945054 10.1371/journal.pbio.3000599 PMC6964821

[60] Hernandez-Segura A, de Jong TV, Melov S, Guryev V, Campisi J, Demaria M. Unmasking transcriptional heterogeneity in senescent cells. Curr Biol. 2017;27(17):2652‐2660.e4. 28844647 10.1016/j.cub.2017.07.033 PMC5788810

[61] Wiley CD, Flynn JM, Morrissey C, et al Analysis of individual cells identifies cell-to-cell variability following induction of cellular senescence. Aging Cell. 2017;16(5):1043‐1050. 28699239 10.1111/acel.12632 PMC5595671

[62] Jochems F, Thijssen B, De Conti G, et al The cancer SENESCopedia: a delineation of cancer cell senescence. Cell Rep. 2021;36(4):109441. 34320349 10.1016/j.celrep.2021.109441 PMC8333195

[63] Tripathi U, Misra A, Tchkonia T, Kirkland JL. Impact of senescent cell subtypes on tissue dysfunction and repair: importance and research questions. Mech Ageing Dev. 2021;198:111548. 34352325 10.1016/j.mad.2021.111548 PMC8373827

[64] Hudgins AD, Tazearslan C, Tare A, Zhu Y, Huffman D, Suh Y. Age- and tissue-specific expression of senescence biomarkers in mice. Front Genet. 2018;9:59. 29527222 10.3389/fgene.2018.00059 PMC5829053

[65] Kennedy BK, Berger SL, Brunet A, et al Geroscience: linking aging to chronic disease. Cell. 2014;159(4):709‐713. 25417146 10.1016/j.cell.2014.10.039 PMC4852871

[66] Inouye SK, Studenski S, Tinetti ME, Kuchel GA. Geriatric syndromes: clinical, research, and policy implications of a core geriatric concept. J Am Geriatr Soc. 2007;55(5):780‐791. 17493201 10.1111/j.1532-5415.2007.01156.x PMC2409147

[67] Hadley EC, Kuchel GA, Newman AB. Report: NIA workshop on measures of physiologic resiliencies in human aging. J Gerontol A Biol Sci Med Sci. 2017;72(7):980‐990. 28475732 10.1093/gerona/glx015 PMC5861884

[68] Raffaele M, Vinciguerra M. The costs and benefits of senotherapeutics for human health. Lancet Healthy Longev. 2022;3(1):e67‐e77. 36098323 10.1016/S2666-7568(21)00300-7

[69] López-Otín C, Blasco MA, Partridge L, Serrano M, Kroemer G. The hallmarks of aging. Cell. 2013;153(6):1194‐1217. 23746838 10.1016/j.cell.2013.05.039 PMC3836174

[70] Sinclair AJ, Conroy SP, Bayer AJ. Impact of diabetes on physical function in older people. Diabetes Care. 2008;31(2):233‐235. 18024850 10.2337/dc07-1784

[71] Corriere M, Rooparinesingh N, Kalyani RR. Epidemiology of diabetes and diabetes complications in the elderly: an emerging public health burden. Curr Diab Rep. 2013;13(6):805‐813. 24018732 10.1007/s11892-013-0425-5 PMC3856245

[72] Kalyani RR, Metter EJ, Ramachandran R, Chia CW, Saudek CD, Ferrucci L. Glucose and insulin measurements from the oral glucose tolerance test and relationship to muscle mass. J Gerontol A Biol Sci Med Sci. 2012;67(1):74‐81. 21350243 10.1093/gerona/glr022 PMC3260481

[73] Dimri GP, Lee X, Basile G, et al A biomarker that identifies senescent human cells in culture and in aging skin in vivo. Proc Natl Acad Sci U S A. 1995;92(20):9363‐9367. 7568133 10.1073/pnas.92.20.9363 PMC40985

[74] González-Gualda E, Baker AG, Fruk L, Muñoz-Espín D. A guide to assessing cellular senescence in vitro and in vivo. FEBS J. 2021;288(1):56‐80. 32961620 10.1111/febs.15570

[75] Zhu Y, Tchkonia T, Pirtskhalava T, et al The Achilles’ heel of senescent cells: from transcriptome to senolytic drugs. Aging Cell. 2015;14(4):644‐658. 25754370 10.1111/acel.12344 PMC4531078

[76] Swanson EC, Manning B, Zhang H, Lawrence JB. Higher-order unfolding of satellite heterochromatin is a consistent and early event in cell senescence. J Cell Biol. 2013;203(6):929‐942. 24344186 10.1083/jcb.201306073 PMC3871423

[77] Narita M, Nũnez S, Heard E, et al Rb-mediated heterochromatin formation and silencing of E2F target genes during cellular senescence. Cell. 2003;113(6):703‐716. 12809602 10.1016/s0092-8674(03)00401-x

[78] Farr JN, Fraser DG, Wang H, et al Identification of senescent cells in the bone microenvironment. J Bone Miner Res. 2016;31(11):1920‐1929. 27341653 10.1002/jbmr.2892 PMC5289710

[79] Iske J, Seyda M, Heinbokel T, et al Senolytics prevent mt-DNA-induced inflammation and promote the survival of aged organs following transplantation. Nat Commun. 2020;11(1):4289. 32855397 10.1038/s41467-020-18039-x PMC7453018

[80] Papaconstantinou J . Insulin/IGF-1 and ROS signaling pathway cross-talk in aging and longevity determination. Mol Cell Endocrinol. 2009;299(1):89‐100. 19103250 10.1016/j.mce.2008.11.025 PMC2873688

[81] Daneshgar N, Dai DF. ROS, Klotho and mTOR in cardiorenal aging. Aging (Albany NY). 2020;12(20):19830‐19831. 33125344 10.18632/aging.104209 PMC7655185

[82] Wu C, Ma X, Zhou Y, Liu Y, Shao Y, Wang Q. Klotho restraining Egr1/TLR4/mTOR axis to reducing the expression of fibrosis and inflammatory cytokines in high glucose cultured rat mesangial cells. Exp Clin Endocrinol Diabetes. 2019;127(9):630‐640. 29890551 10.1055/s-0044-101601

[83] Rakugi H, Matsukawa N, Ishikawa K, et al Anti-oxidative effect of Klotho on endothelial cells through cAMP activation. Endocrine. 2007;31(1):82‐87. 17709902 10.1007/s12020-007-0016-9

[84] Xie L, Wang Y, Li Q, et al The HIF-1α/p53/miRNA-34a/Klotho axis in retinal pigment epithelial cells promotes subretinal fibrosis and exacerbates choroidal neovascularization. J Cell Mol Med. 2021;25(3):1700‐1711. 33438362 10.1111/jcmm.16272 PMC7875902

[85] Kuro OM . The Klotho proteins in health and disease. Nat Rev Nephrol. 2019;15(1):27‐44. 30455427 10.1038/s41581-018-0078-3

[86] Zhu Y, Prata L, Gerdes EOW, et al Orally-active, clinically-translatable senolytics restore α-Klotho in mice and humans. EBioMedicine. 2022;77:103912. 35292270 10.1016/j.ebiom.2022.103912 PMC9034457

[87] Munk R, Panda AC, Grammatikakis I, Gorospe M, Abdelmohsen K. Senescence-associated microRNAs. Int Rev Cell Mol Biol. 2017;334:177‐205. 28838538 10.1016/bs.ircmb.2017.03.008 PMC8436595

[88] Suh N . MicroRNA controls of cellular senescence. BMB Rep. 2018;51(10):493‐499. 30269742 10.5483/BMBRep.2018.51.10.209 PMC6235093

[89] Tanaka Y, Takahashi A. Senescence-associated extracellular vesicle release plays a role in senescence-associated secretory phenotype (SASP) in age-associated diseases. J Biochem. 2021;169(2):147‐153. 33002139 10.1093/jb/mvaa109

[90] Misawa T, Hitomi K, Miyata K, et al Identification of novel senescent markers in small extracellular vesicles. Int J Mol Sci. 2023;24(3):2421. 36768745 10.3390/ijms24032421 PMC9916821

[91] Faraonio R, Salerno P, Passaro F, et al A set of miRNAs participates in the cellular senescence program in human diploid fibroblasts. Cell Death Differ. 2012;19(4):713‐721. 22052189 10.1038/cdd.2011.143 PMC3307984

[92] Mori MA, Raghavan P, Thomou T, et al Role of microRNA processing in adipose tissue in stress defense and longevity. Cell Metab. 2012;16(3):336‐347. 22958919 10.1016/j.cmet.2012.07.017 PMC3461823

[93] Gorgoulis V, Adams PD, Alimonti A, et al Cellular senescence: defining a path forward. Cell. 2019;179(4):813‐827. 31675495 10.1016/j.cell.2019.10.005

[94] Biran A, Zada L, Abou Karam P, et al Quantitative identification of senescent cells in aging and disease. Aging Cell. 2017;16(4):661‐671. 28455874 10.1111/acel.12592 PMC5506427

[95] Hickson LJ, Langhi Prata LGP, Bobart SA, et al Senolytics decrease senescent cells in humans: preliminary report from a clinical trial of Dasatinib plus Quercetin in individuals with diabetic kidney disease. EBioMedicine. 2019;47:446‐456. 31542391 10.1016/j.ebiom.2019.08.069 PMC6796530

[96] Saul D, Kosinsky RL, Atkinson EJ, et al A new gene set identifies senescent cells and predicts senescence-associated pathways across tissues. Nat Commun. 2022;13(1):4827. 35974106 10.1038/s41467-022-32552-1 PMC9381717

[97] Liu Y, Sanoff HK, Cho H, et al Expression of p16(INK4a) in peripheral blood T-cells is a biomarker of human aging. Aging Cell. 2009;8(4):439‐448. 19485966 10.1111/j.1474-9726.2009.00489.x PMC2752333

[98] Englund DA, Sakamoto AE, Fritsche CM, et al Exercise reduces circulating biomarkers of cellular senescence in humans. Aging Cell. 2021;20(7):e13415. 34101960 10.1111/acel.13415 PMC8282238

[99] CDC . National diabetes statistics report. Accessed April 5, 2024. https://www.cdc.gov/diabetes/data/statistics-report/index.html

[100] Kalyani RR, Egan JM. Diabetes and altered glucose metabolism with aging. Endocrinol Metab Clin North Am. 2013;42(2):333‐347. 23702405 10.1016/j.ecl.2013.02.010 PMC3664017

[101] Hauner H . Secretory factors from human adipose tissue and their functional role. Proc Nutr Soc. 2005;64(2):163‐169. 15960861 10.1079/pns2005428

[102] Halberg N, Wernstedt-Asterholm I, Scherer PE. The adipocyte as an endocrine cell. Endocrinol Metab Clin North Am. 2008;37(3):753‐768, x-xi. 18775362 10.1016/j.ecl.2008.07.002 PMC2659415

[103] Coelho M, Oliveira T, Fernandes R. Biochemistry of adipose tissue: an endocrine organ. Arch Med Sci. 2013;9(2):191‐200. 23671428 10.5114/aoms.2013.33181 PMC3648822

[104] Kirkland JL, Tchkonia T, Pirtskhalava T, Han J, Karagiannides I. Adipogenesis and aging: does aging make fat go MAD? Exp Gerontol. 2002;37(6):757‐767. 12175476 10.1016/s0531-5565(02)00014-1

[105] Minamino T, Orimo M, Shimizu I, et al A crucial role for adipose tissue p53 in the regulation of insulin resistance. Nat Med. 2009;15(9):1082‐1087. 19718037 10.1038/nm.2014

[106] Villaret A, Galitzky J, Decaunes P, et al Adipose tissue endothelial cells from obese human subjects: differences among depots in angiogenic, metabolic, and inflammatory gene expression and cellular senescence. Diabetes. 2010;59(11):2755‐2763. 20713685 10.2337/db10-0398 PMC2963533

[107] Dhirachaikulpanich D, Lagger C, Chatsirisupachai K, de Magalhães JP, Paraoan L. Intercellular communication analysis of the human retinal pigment epithelial and choroidal cells predicts pathways associated with aging, cellular senescence and age-related macular degeneration. Front Aging Neurosci. 2022;14:1016293. 36408112 10.3389/fnagi.2022.1016293 PMC9669800

[108] Chae JB, Park CW, Lee HM, et al Accelerated aging phenotypes in the retinal pigment epithelium of Zmpste24-deficient mice. Biochem Biophys Res Commun. 2022;632:62‐68. 36201882 10.1016/j.bbrc.2022.09.061

[109] Russell SJ, Kahn CR. Endocrine regulation of ageing. Nat Rev Mol Cell Biol. 2007;8(9):681‐691. 17684529 10.1038/nrm2234

[110] Kimura KD, Tissenbaum HA, Liu Y, Ruvkun G. daf-2, an insulin receptor-like gene that regulates longevity and diapause in Caenorhabditis elegans. Science. 1997;277(5328):942‐946. 9252323 10.1126/science.277.5328.942

[111] Kenyon C, Chang J, Gensch E, Rudner A, Tabtiang R. A C. elegans mutant that lives twice as long as wild type. Nature. 1993;366(6454):461‐464. 8247153 10.1038/366461a0

[112] Minamino T, Komuro I. Vascular cell senescence: contribution to atherosclerosis. Circ Res. 2007;100(1):15‐26. 17204661 10.1161/01.RES.0000256837.40544.4a

[113] Hwangbo DS, Gershman B, Tu MP, Palmer M, Tatar M. Drosophila dFOXO controls lifespan and regulates insulin signalling in brain and fat body. Nature. 2004;429(6991):562‐566. 15175753 10.1038/nature02549

[114] Giannakou ME, Goss M, Jünger MA, Hafen E, Leevers SJ, Partridge L. Long-lived Drosophila with overexpressed dFOXO in adult fat body. Science. 2004;305(5682):361. 15192154 10.1126/science.1098219

[115] Fabrizio P, Pozza F, Pletcher SD, Gendron CM, Longo VD. Regulation of longevity and stress resistance by Sch9 in yeast. Science. 2001;292(5515):288‐290. 11292860 10.1126/science.1059497

[116] Fabrizio P, Liou LL, Moy VN, et al SOD2 functions downstream of Sch9 to extend longevity in yeast. Genetics. 2003;163(1):35‐46. 12586694 10.1093/genetics/163.1.35 PMC1462415

[117] Clancy DJ, Gems D, Harshman LG, et al Extension of life-span by loss of CHICO, a Drosophila insulin receptor substrate protein. Science. 2001;292(5514):104‐106. 11292874 10.1126/science.1057991

[118] Holzenberger M, Dupont J, Ducos B, et al IGF-1 receptor regulates lifespan and resistance to oxidative stress in mice. Nature. 2003;421(6919):182‐187. 12483226 10.1038/nature01298

[119] Blüher M, Kahn BB, Kahn CR. Extended longevity in mice lacking the insulin receptor in adipose tissue. Science. 2003;299(5606):572‐574. 12543978 10.1126/science.1078223

[120] Taguchi A, Wartschow LM, White MF. Brain IRS2 signaling coordinates life span and nutrient homeostasis. Science. 2007;317(5836):369‐372. 17641201 10.1126/science.1142179

[121] Aguiar-Oliveira MH, Bartke A. Growth hormone deficiency: health and longevity. Endocr Rev. 2019;40(2):575‐601. 30576428 10.1210/er.2018-00216 PMC6416709

[122] Colman RJ, Anderson RM, Johnson SC, et al Caloric restriction delays disease onset and mortality in rhesus monkeys. Science. 2009;325(5937):201‐204. 19590001 10.1126/science.1173635 PMC2812811

[123] Ramsey JJ, Colman RJ, Binkley NC, et al Dietary restriction and aging in rhesus monkeys: the university of Wisconsin study. Exp Gerontol. 2000;35(9-10):1131‐1149. 11113597 10.1016/s0531-5565(00)00166-2

[124] Gresl TA, Colman RJ, Roecker EB, et al Dietary restriction and glucose regulation in aging rhesus monkeys: a follow-up report at 8.5yr. Am J Physiol Endocrinol Metab. 2001;281(4):E757‐E765. 11551852 10.1152/ajpendo.2001.281.4.E757

[125] Roth GS, Lane MA, Ingram DK, et al Biomarkers of caloric restriction may predict longevity in humans. Science. 2002;297(5582):811. 12161648 10.1126/science.1071851

[126] Ben-Avraham D, Govindaraju DR, Budagov T, et al The GH receptor exon 3 deletion is a marker of male-specific exceptional longevity associated with increased GH sensitivity and taller stature. Sci Adv. 2017;3(6):e1602025. 28630896 10.1126/sciadv.1602025 PMC5473676

[127] van der Spoel E, Jansen SW, Akintola AA, et al Growth hormone secretion is diminished and tightly controlled in humans enriched for familial longevity. Aging Cell. 2016;15(6):1126‐1131. 27605408 10.1111/acel.12519 PMC6398524

[128] Chaib S, Tchkonia T, Kirkland JL. Cellular senescence and senolytics: the path to the clinic. Nat Med. 2022;28(8):1556‐1568. 35953721 10.1038/s41591-022-01923-y PMC9599677

[129] Tchkonia T, Palmer AK, Kirkland JL. New horizons: novel approaches to enhance healthspan through targeting cellular senescence and related aging mechanisms. J Clin Endocrinol Metab. 2021;106(3):e1481‐e1487. 33155651 10.1210/clinem/dgaa728 PMC7947756

[130] Conover CA, Bale LK. Senescence induces proteolytically-active PAPP-A secretion and association with extracellular vesicles in human pre-adipocytes. Exp Gerontol. 2022;172:112070. 36549546 10.1016/j.exger.2022.112070 PMC9868105

[131] Conover CA . Key questions and answers about pregnancy-associated plasma protein-A. Trends Endocrinol Metab. 2012;23(5):242‐249. 22463950 10.1016/j.tem.2012.02.008 PMC3348390

[132] Bale LK, West SA, Conover CA. Inducible knockdown of pregnancy-associated plasma protein-A gene expression in adult female mice extends life span. Aging Cell. 2017;16(4):895‐897. 28600811 10.1111/acel.12624 PMC5506424

[133] Conover CA, Bale LK, Marler RJ. Pregnancy-associated plasma protein-A deficiency improves survival of mice on a high fat diet. Exp Gerontol. 2015;70:131‐134. 26325589 10.1016/j.exger.2015.08.007 PMC4600682

[134] Conover CA, Harstad SL, Tchkonia T, Kirkland JL. Preferential impact of pregnancy-associated plasma protein-A deficiency on visceral fat in mice on high-fat diet. Am J Physiol Endocrinol Metab. 2013;305(9):E1145‐E1153. 24045868 10.1152/ajpendo.00405.2013 PMC3840208

[135] Bian A, Ma Y, Zhou X, et al Association between sarcopenia and levels of growth hormone and insulin-like growth factor-1 in the elderly. BMC Musculoskelet Disord. 2020;21(1):214. 32264885 10.1186/s12891-020-03236-y PMC7140321

[136] Ascenzi F, Barberi L, Dobrowolny G, et al Effects of IGF-1 isoforms on muscle growth and sarcopenia. Aging Cell. 2019;18(3):e12954. 30953403 10.1111/acel.12954 PMC6516183

[137] Chakravarthy MV, Davis BS, Booth FW. IGF-I restores satellite cell proliferative potential in immobilized old skeletal muscle. J Appl Physiol (1985). 2000;89(4):1365‐1379. 11007571 10.1152/jappl.2000.89.4.1365

[138] Latres E, Amini AR, Amini AA, et al Insulin-like growth factor-1 (IGF-1) inversely regulates atrophy-induced genes via the phosphatidylinositol 3-kinase/Akt/mammalian target of rapamycin (PI3K/Akt/mTOR) pathway. J Biol Chem. 2005;280(4):2737‐2744. 15550386 10.1074/jbc.M407517200

[139] O’Neill BT, Lee KY, Klaus K, et al Insulin and IGF-1 receptors regulate FoxO-mediated signaling in muscle proteostasis. J Clin Invest. 2016;126(9):3433‐3446. 27525440 10.1172/JCI86522 PMC5004956

[140] Rudman D, Feller AG, Nagraj HS, et al Effects of human growth hormone in men over 60 years old. N Engl J Med. 1990;323(1):1‐6. 2355952 10.1056/NEJM199007053230101

[141] Beigienė A, Petruševičienė D, Barasaitė V, Kubilius R, Macijauskienė J. Frailty and different exercise interventions to improve gait speed in older adults after acute coronary syndrome. Medicina (Kaunas). 2021;57(12):1344. 34946289 10.3390/medicina57121344 PMC8705993

[142] Jamali T, Raasikh T, Bustamante G, et al Outcomes of exercise interventions in patients with advanced liver disease: a systematic review of randomized clinical trials. Am J Gastroenterol. 2022;117(10):1614‐1620. 35973182 10.14309/ajg.0000000000001883

[143] Flor-Rufino C, Barrachina-Igual J, Pérez-Ros P, Pablos-Monzó A, Martínez-Arnau FM. Resistance training of peripheral muscles benefits respiratory parameters in older women with sarcopenia: randomized controlled trial. Arch Gerontol Geriatr. 2022;104:104799. 36070636 10.1016/j.archger.2022.104799

[144] Swales B, Ryde GC, Whittaker AC. A randomized controlled feasibility trial evaluating a resistance training intervention with frail older adults in residential care: the keeping active in residential elderly trial. J Aging Phys Act. 2022;30(3):364‐388. 34510020 10.1123/japa.2021-0130

[145] Bolster DR, Jefferson LS, Kimball SR. Regulation of protein synthesis associated with skeletal muscle hypertrophy by insulin-, amino acid- and exercise-induced signalling. Proc Nutr Soc. 2004;63(2):351‐356. 15294054 10.1079/PNS2004355

[146] Ensrud KE . Epidemiology of fracture risk with advancing age. J Gerontol A Biol Sci Med Sci. 2013;68(10):1236‐1242. 23833201 10.1093/gerona/glt092

[147] Sambrook P, Cooper C. Osteoporosis. Lancet. 2006;367(9527):2010‐2018. 16782492 10.1016/S0140-6736(06)68891-0

[148] MacLaughlin J, Holick MF. Aging decreases the capacity of human skin to produce vitamin D3. J Clin Invest. 1985;76(4):1536‐1538. 2997282 10.1172/JCI112134 PMC424123

[149] Franco AC, Aveleira C, Cavadas C. Skin senescence: mechanisms and impact on whole-body aging. Trends Mol Med. 2022;28(2):97‐109. 35012887 10.1016/j.molmed.2021.12.003

[150] Farr JN, Xu M, Weivoda MM, et al Targeting cellular senescence prevents age-related bone loss in mice. Nat Med. 2017;23(9):1072‐1079. 28825716 10.1038/nm.4385 PMC5657592

[151] Khosla S, Farr JN, Tchkonia T, Kirkland JL. The role of cellular senescence in ageing and endocrine disease. Nat Rev Endocrinol. 2020;16(5):263‐275. 32161396 10.1038/s41574-020-0335-y PMC7227781

[152] Yang SB, Tien AC, Boddupalli G, Xu AW, Jan YN, Jan LY. Rapamycin ameliorates age-dependent obesity associated with increased mTOR signaling in hypothalamic POMC neurons. Neuron. 2012;75(3):425‐436. 22884327 10.1016/j.neuron.2012.03.043 PMC3467009

[153] Veldhuis JD . Changes in pituitary function with ageing and implications for patient care. Nat Rev Endocrinol. 2013;9(4):205‐215. 23438832 10.1038/nrendo.2013.38 PMC3920108

[154] Morimoto N, Kawakami F, Makino S, Chihara K, Hasegawa M, Ibata Y. Age-related changes in growth hormone releasing factor and somatostatin in the rat hypothalamus. Neuroendocrinology. 1988;47(5):459‐464. 2899847 10.1159/000124950

[155] Farhy LS, Veldhuis JD. Deterministic construct of amplifying actions of ghrelin on pulsatile growth hormone secretion. Am J Physiol Regul Integr Comp Physiol. 2005;288(6):R1649‐R1663. 15718392 10.1152/ajpregu.00451.2004

[156] Iranmanesh A, South S, Liem AY, et al Unequal impact of age, percentage body fat, and serum testosterone concentrations on the somatotrophic, IGF-I, and IGF-binding protein responses to a three-day intravenous growth hormone-releasing hormone pulsatile infusion in men. Eur J Endocrinol. 1998;139(1):59‐71. 9703380 10.1530/eje.0.1390059

[157] Gentili A, Mulligan T, Godschalk M, et al Unequal impact of short-term testosterone repletion on the somatotropic axis of young and older men. J Clin Endocrinol Metab. 2002;87(2):825‐834. 11836328 10.1210/jcem.87.2.8222

[158] Moiseeva V, Cisneros A, Sica V, et al Senescence atlas reveals an aged-like inflamed niche that blunts muscle regeneration. Nature. 2022;613(7942):169‐178. 36544018 10.1038/s41586-022-05535-x PMC9812788

[159] Anghel L, Baroiu L, Popazu CR, et al Benefits and adverse events of melatonin use in the elderly (review). Exp Ther Med. 2022;23(3):219. 35126722 10.3892/etm.2022.11142 PMC8796282

[160] Kondratov RV, Kondratova AA, Gorbacheva VY, Vykhovanets OV, Antoch MP. Early aging and age-related pathologies in mice deficient in BMAL1, the core componentof the circadian clock. Genes Dev. 2006;20(14):1868‐1873. 16847346 10.1101/gad.1432206 PMC1522083

[161] Antoch MP, Gorbacheva VY, Vykhovanets O, et al Disruption of the circadian clock due to the Clock mutation has discrete effects on aging and carcinogenesis. Cell Cycle. 2008;7(9):1197‐1204. 18418054 10.4161/cc.7.9.5886 PMC2744375

[162] Dubrovsky YV, Samsa WE, Kondratov RV. Deficiency of circadian protein CLOCK reduces lifespan and increases age-related cataract development in mice. Aging (Albany NY). 2010;2(12):936‐944. 21149897 10.18632/aging.100241 PMC3034182

[163] Carroll JE, Irwin MR, Levine M, et al Epigenetic aging and immune senescence in women with insomnia symptoms: findings from the women's health initiative study. Biol Psychiatry. 2017;81(2):136‐144. 27702440 10.1016/j.biopsych.2016.07.008 PMC5536960

[164] Carroll JE, Olmstead R, Cole SW, Breen EC, Arevalo JM, Irwin MR. Remission of insomnia in older adults treated with cognitive behavioral therapy for insomnia (CBT-I) reduces p16(INK4a) gene expression in peripheral blood: secondary outcome analysis from a randomized clinical trial. Geroscience. 2023;45(4):2325‐2335. 36849678 10.1007/s11357-023-00741-5 PMC10651570

[165] Carreras A, Zhang SX, Peris E, et al Chronic sleep fragmentation induces endothelial dysfunction and structural vascular changes in mice. Sleep. 2014;37(11):1817‐1824. 25364077 10.5665/sleep.4178 PMC4196065

[166] Ezzat S, Asa SL, Couldwell WT, et al The prevalence of pituitary adenomas: a systematic review. Cancer. 2004;101(3):613‐619. 15274075 10.1002/cncr.20412

[167] Kuilman T, Michaloglou C, Vredeveld LC, et al Oncogene-induced senescence relayed by an interleukin-dependent inflammatory network. Cell. 2008;133(6):1019‐1031. 18555778 10.1016/j.cell.2008.03.039

[168] Manojlovic-Gacic E, Skender-Gazibara M, Popovic V, et al Oncogene-induced senescence in pituitary adenomas--an immunohistochemical study. Endocr Pathol. 2016;27(1):1‐11. 26573928 10.1007/s12022-015-9405-4

[169] Hansen KR, Knowlton NS, Thyer AC, Charleston JS, Soules MR, Klein NA. A new model of reproductive aging: the decline in ovarian non-growing follicle number from birth to menopause. Hum Reprod. 2008;23(3):699‐708. 18192670 10.1093/humrep/dem408

[170] El Khoudary SR, Aggarwal B, Beckie TM, et al Menopause transition and cardiovascular disease risk: implications for timing of early prevention: a scientific statement from the American Heart Association. Circulation. 2020;142(25):e506‐e532. 33251828 10.1161/CIR.0000000000000912

[171] Alberico HC, Woods DC. Role of granulosa cells in the aging ovarian landscape: a focus on mitochondrial and metabolic function. Front Physiol. 2021;12:800739. 35153812 10.3389/fphys.2021.800739 PMC8829508

[172] Secomandi L, Borghesan M, Velarde M, Demaria M. The role of cellular senescence in female reproductive aging and the potential for senotherapeutic interventions. Hum Reprod Update. 2022;28(2):172‐189. 34918084 10.1093/humupd/dmab038 PMC8888999

[173] Krishnamurthy J, Torrice C, Ramsey MR, et al Ink4a/Arf expression is a biomarker of aging. J Clin Invest. 2004;114(9):1299‐1307. 15520862 10.1172/JCI22475 PMC524230

[174] Merz SE, Klopfleisch R, Breithaupt A, Gruber AD. Aging and senescence in canine testes. Vet Pathol. 2019;56(5):715‐724. 31060479 10.1177/0300985819843683

[175] Kusama K, Yamauchi N, Yoshida K, Azumi M, Yoshie M, Tamura K. Senolytic treatment modulates decidualization in human endometrial stromal cells. Biochem Biophys Res Commun. 2021;571:174‐180. 34330061 10.1016/j.bbrc.2021.07.075

[176] Liu F, Wan Q, Liu P, Miao D, Dai X, Chen L. Loss of p16 does not protect against premature ovarian insufficiency caused by alkylating agents. BMC Pregnancy Childbirth. 2023;23(1):151. 36890528 10.1186/s12884-023-05476-x PMC9993597

[177] Nair KS, Rizza RA, O’Brien P, et al DHEA in elderly women and DHEA or testosterone in elderly men. N Engl J Med. 2006;355(16):1647‐1659. 17050889 10.1056/NEJMoa054629

[178] Enomoto M, Adachi H, Fukami A, et al Serum dehydroepiandrosterone sulfate levels predict longevity in men: 27-year follow-up study in a community-based cohort (Tanushimaru study). J Am Geriatr Soc. 2008;56(6):994‐998. 18422949 10.1111/j.1532-5415.2008.01692.x

[179] Wierman ME, Kiseljak-Vassiliades K. Should dehydroepiandrosterone be administered to women? J Clin Endocrinol Metab. 2022;107(6):1679‐1685. 35254428 10.1210/clinem/dgac130 PMC9113789

[180] Tatomir A, Micu C, Crivii C. The impact of stress and glucocorticoids on memory. Clujul Med. 2014;87(1):3‐6. 26527987 10.15386/cjm.2014.8872.871.at1cm2 PMC4462413

[181] Guan R, Yang C, Zhang J, Wang J, Chen R, Su P. Dehydroepiandrosterone alleviates hypoxia-induced learning and memory dysfunction by maintaining synaptic homeostasis. CNS Neurosci Ther. 2022;28(9):1339‐1350. 35703574 10.1111/cns.13869 PMC9344085

[182] Kau MM, Chen JJ, Wang SW, Cho WL, Wang PS. Age-related impairment of aldosterone secretion in zona glomerulosa cells of ovariectomized rats. J Investig Med. 1999;47(8):425‐432. 10510595

[183] Gao X, Li F, Liu B, Wang Y, Wang Y, Zhou H. Cellular senescence in adrenocortical biology and its disorders. Cells. 2021;10(12):3474. 34943980 10.3390/cells10123474 PMC8699888

[184] De Stefano MA, Porcelli T, Ambrosio R, et al Type 2 deiodinase is expressed in anaplastic thyroid carcinoma and its inhibition causes cell senescence. Endocr Relat Cancer. 2023;30(5):e230016. 36877008 10.1530/ERC-23-0016 PMC10160549

[185] Percheron G, Hogrel JY, Denot-Ledunois S, et al Effect of 1-year oral administration of dehydroepiandrosterone to 60- to 80-year-old individuals on muscle function and cross-sectional area: a double-blind placebo-controlled trial. Arch Intern Med. 2003;163(6):720‐727. 12639206 10.1001/archinte.163.6.720

[186] Bowers J, Terrien J, Clerget-Froidevaux MS, et al Thyroid hormone signaling and homeostasis during aging. Endocr Rev. 2013;34(4):556‐589. 23696256 10.1210/er.2012-1056

[187] Franceschi C, Ostan R, Mariotti S, Monti D, Vitale G. The aging thyroid: a reappraisal within the geroscience integrated perspective. Endocr Rev. 2019;40(5):1250‐1270. 31074798 10.1210/er.2018-00170

[188] Buffenstein R, Pinto M. Endocrine function in naturally long-living small mammals. Mol Cell Endocrinol. 2009;299(1):101‐111. 18674586 10.1016/j.mce.2008.04.021 PMC4399555

[189] Zambrano A, García-Carpizo V, Gallardo ME, et al The thyroid hormone receptor β induces DNA damage and premature senescence. J Cell Biol. 2014;204(1):129‐146. 24395638 10.1083/jcb.201305084 PMC3882795

[190] Chini CCS, Peclat TR, Warner GM, et al CD38 ecto-enzyme in immune cells is induced during aging and regulates NAD(+) and NMN levels. Nat Metab. 2020;2(11):1284‐1304. 33199925 10.1038/s42255-020-00298-z PMC8752031

[191] Lagnado A, Leslie J, Ruchaud-Sparagano MH, et al Neutrophils induce paracrine telomere dysfunction and senescence in ROS-dependent manner. EMBO J. 2021;40(9):e106048. 33764576 10.15252/embj.2020106048 PMC8090854

[192] Kammori M, Nakamura K, Kawahara M, Mimura Y, Kaminishi M, Takubo K. Telomere shortening with aging in human thyroid and parathyroid tissue. Exp Gerontol. 2002;37(4):513‐521. 11830354 10.1016/s0531-5565(01)00178-4

[193] Bates JN, Kohn TP, Pastuszak AW. Effect of thyroid hormone derangements on sexual function in men and women. Sex Med Rev. 2020;8(2):217‐230. 30458985 10.1016/j.sxmr.2018.09.005 PMC6525090

[194] Meng Z, Liu M, Zhang Q, et al Gender and age impact on the association between thyroid-stimulating hormone and serum lipids. Medicine (Baltimore). 2015;94(49):e2186. 26656346 10.1097/MD.0000000000002186 PMC5008491

[195] Riis J, Pedersen KM, Danielsen MB, et al Long-term iodine nutrition is associated with longevity in older adults: a 20 years’ follow-up of the Randers-Skagen study. Br J Nutr. 2021;125(3):260‐265. 32378500 10.1017/S0007114520001592

[196] Tolu F, Palermo M, Dore MP, et al Association of endemic goitre and exceptional longevity in Sardinia: evidence from an ecological study. Eur J Ageing. 2019;16(4):405‐414. 31798366 10.1007/s10433-019-00510-4 PMC6857103

[197] Jansen SW, Akintola AA, Roelfsema F, et al Human longevity is characterised by high thyroid stimulating hormone secretion without altered energy metabolism. Sci Rep. 2015;5(1):11525. 26089239 10.1038/srep11525 PMC4473605

[198] Maniakas A, Dadu R, Busaidy NL, et al Evaluation of overall survival in patients with anaplastic thyroid carcinoma, 2000–2019. JAMA Oncol. 2020;6(9):1397‐1404. 32761153 10.1001/jamaoncol.2020.3362 PMC7411939

[199] Prasanna PG, Citrin DE, Hildesheim J, et al Therapy-Induced senescence: opportunities to improve anticancer therapy. J Natl Cancer Inst. 2021;113(10):1285‐1298. 33792717 10.1093/jnci/djab064 PMC8486333

[200] Guida JL, Agurs-Collins T, Ahles TA, et al Strategies to prevent or remediate cancer and treatment-related aging. J Natl Cancer Inst. 2021;113(2):112‐122. 32348501 10.1093/jnci/djaa060 PMC7850536

[201] Chalcraft JR, Cardinal LM, Wechsler PJ, et al Vitamin D synthesis following a single bout of sun exposure in older and younger men and women. Nutrients. 2020;12(8):2237. 32727044 10.3390/nu12082237 PMC7468901

[202] Wacker M, Holick MF. Sunlight and vitamin D: a global perspective for health. Dermatoendocrinol. 2013;5(1):51‐108. 24494042 10.4161/derm.24494 PMC3897598

[203] Campisi J . The role of cellular senescence in skin aging. J Investig Dermatol Symp Proc. 1998;3(1):1‐5. 9732048

[204] Wyles SP, Tchkonia T, Kirkland JL. Targeting cellular senescence for age-related diseases: path to clinical translation. Plast Reconstr Surg. 2022;150:20s‐26s. 10.1097/PRS.0000000000009669 PMC9529239 36170432

[205] Gonçalves de Carvalho CM, Ribeiro SM. Aging, low-grade systemic inflammation and vitamin D: a mini-review. Eur J Clin Nutr. 2017;71(4):434‐440. 27677370 10.1038/ejcn.2016.177

[206] de Jongh RT, van Schoor NM, Lips P. Changes in vitamin D endocrinology during aging in adults. Mol Cell Endocrinol. 2017;453:144‐150. 28602863 10.1016/j.mce.2017.06.005

[207] Hill TR, Granic A, Aspray TJ. Vitamin D and ageing. Subcell Biochem. 2018;90:191‐220. 30779011 10.1007/978-981-13-2835-0_8

[208] Gallagher JC . Vitamin D and aging. Endocrinol Metab Clin North Am. 2013;42(2):319‐332. 23702404 10.1016/j.ecl.2013.02.004 PMC3782116

[209] Amrein K, Scherkl M, Hoffmann M, et al Vitamin D deficiency 2.0: an update on the current status worldwide. Eur J Clin Nutr. 2020;74(11):1498‐1513. 31959942 10.1038/s41430-020-0558-y PMC7091696

[210] Vranić L, Mikolašević I, Milić S. Vitamin D deficiency: consequence or cause of obesity? Medicina (Kaunas). 2019;55(9):541. 31466220 10.3390/medicina55090541 PMC6780345

[211] Lips P, van Schoor NM. The effect of vitamin D on bone and osteoporosis. Best Pract Res Clin Endocrinol Metab. 2011;25(4):585‐591. 21872800 10.1016/j.beem.2011.05.002

[212] Richards JB, Valdes AM, Gardner JP, et al Higher serum vitamin D concentrations are associated with longer leukocyte telomere length in women. Am J Clin Nutr. 2007;86(5):1420‐1425. 17991655 10.1093/ajcn/86.5.1420 PMC2196219

[213] Chen L, Yang R, Qiao W, et al 1,25-Dihydroxyvitamin d exerts an antiaging role by activation of Nrf2-antioxidant signaling and inactivation of p16/p53-senescence signaling. Aging Cell. 2019;18(3):e12951. 30907059 10.1111/acel.12951 PMC6516172

[214] Barbouti A, Evangelou K, Pateras IS, et al In situ evidence of cellular senescence in thymic epithelial cells (TECs) during human thymic involution. Mech Ageing Dev. 2019;177:88‐90. 29490231 10.1016/j.mad.2018.02.005

[215] Pan XH, Lin QK, Yao X, et al Umbilical cord mesenchymal stem cells protect thymus structure and function in aged C57 mice by downregulating aging-related genes and upregulating autophagy- and anti-oxidative stress-related genes. Aging (Albany NY). 2020;12(17):16899‐16920. 32924972 10.18632/aging.103594 PMC7521525

[216] Palmer S, Albergante L, Blackburn CC, Newman TJ. Thymic involution and rising disease incidence with age. Proc Natl Acad Sci U S A. 2018;115(8):1883‐1888. 29432166 10.1073/pnas.1714478115 PMC5828591

[217] Aw D, Silva AB, Maddick M, von Zglinicki T, Palmer DB. Architectural changes in the thymus of aging mice. Aging Cell. 2008;7(2):158‐167. 18241323 10.1111/j.1474-9726.2007.00365.x

[218] Thomas R, Wang W, Su D-M. Contributions of age-related thymic involution to immunosenescence and inflammaging. Immun Ageing. 2020;17(1):2. 31988649 10.1186/s12979-020-0173-8 PMC6971920

[219] van der Loo B, Labugger R, Skepper JN, et al Enhanced peroxynitrite formation is associated with vascular aging. J Exp Med. 2000;192(12):1731‐1744. 11120770 10.1084/jem.192.12.1731 PMC2213492

[220] Sato I, Kaji K, Morita I, Nagao M, Murota S-I. Augmentation of endothelin-1, prostacyclin and thromboxane A2 secretion associated with in vitro ageing in cultured human umbilical vein endothelial cells. Mech Ageing Dev. 1993;71(1-2):73‐84. 8309284 10.1016/0047-6374(93)90036-q

[221] Comi P, Chiaramonte R, Maier JA. Senescence-dependent regulation of type 1 plasminogen activator inhibitor in human vascular endothelial cells. Exp Cell Res. 1995;219(1):304‐308. 7628547 10.1006/excr.1995.1232

[222] Garfinkel S, Brown S, Wessendorf JH, Maciag T. Post-transcriptional regulation of interleukin 1 alpha in various strains of young and senescent human umbilical vein endothelial cells. Proc Natl Acad Sci U S A. 1994;91(4):1559‐1563. 8108444 10.1073/pnas.91.4.1559 PMC43199

[223] Minamino T, Miyauchi H, Yoshida T, Ishida Y, Yoshida H, Komuro I. Endothelial cell senescence in human atherosclerosis: role of telomere in endothelial dysfunction. Circulation. 2002;105(13):1541‐1544. 11927518 10.1161/01.cir.0000013836.85741.17

[224] Yokoyama M, Shimizu I, Nagasawa A, et al P53 plays a crucial role in endothelial dysfunction associated with hyperglycemia and ischemia. J Mol Cell Cardiol. 2019;129:105‐117. 30790589 10.1016/j.yjmcc.2019.02.010

[225] Yokoyama M, Okada S, Nakagomi A, et al Inhibition of endothelial p53 improves metabolic abnormalities related to dietary obesity. Cell Rep. 2014;7(5):1691‐1703. 24857662 10.1016/j.celrep.2014.04.046

[226] Suda M, Shimizu I, Katsuumi G, et al Senolytic vaccination improves normal and pathological age-related phenotypes and increases lifespan in progeroid mice. Nat Aging. 2021;1(12):1117‐1126. 37117524 10.1038/s43587-021-00151-2

[227] Kunieda T, Minamino T, Nishi J, et al Angiotensin II induces premature senescence of vascular smooth muscle cells and accelerates the development of atherosclerosis via a p21-dependent pathway. Circulation. 2006;114(9):953‐960. 16908765 10.1161/CIRCULATIONAHA.106.626606

[228] Faget DV, Ren Q, Stewart SA. Unmasking senescence: context-dependent effects of SASP in cancer. Nat Rev Cancer. 2019;19(8):439‐453. 31235879 10.1038/s41568-019-0156-2

[229] Biron-Shental T, Kidron D, Sukenik-Halevy R, et al TERC telomerase subunit gene copy number in placentas from pregnancies complicated with intrauterine growth restriction. Early Hum Dev. 2011;87(2):73‐75. 21168289 10.1016/j.earlhumdev.2010.08.024

[230] Heazell AE, Sharp AN, Baker PN, Crocker IP. Intra-uterine growth restriction is associated with increased apoptosis and altered expression of proteins in the p53 pathway in villous trophoblast. Apoptosis. 2011;16(2):135‐144. 21052841 10.1007/s10495-010-0551-3

[231] Xu M, Pirtskhalava T, Farr JN, et al Senolytics improve physical function and increase lifespan in old age. Nat Med. 2018;24(8):1246‐1256. 29988130 10.1038/s41591-018-0092-9 PMC6082705

[232] Cubro H, Nath KA, Suvakov S, et al Mechanisms of vascular dysfunction in the interleukin-10–deficient murine model of preeclampsia indicate nitric oxide dysregulation. Kidney Int. 2021;99(3):646‐656. 33144212 10.1016/j.kint.2020.09.034 PMC7914163

[233] Suvakov S, Cubro H, White WM, et al Targeting senescence improves angiogenic potential of adipose-derived mesenchymal stem cells in patients with preeclampsia. Biol Sex Differ. 2019;10(1):49. 31521202 10.1186/s13293-019-0263-5 PMC6744626

[234] Suvakov S, Ghamrawi R, Cubro H, et al Epigenetic and senescence markers indicate an accelerated ageing-like state in women with preeclamptic pregnancies. EBioMedicine. 2021;70:103536. 34391091 10.1016/j.ebiom.2021.103536 PMC8365351

[235] Tchkonia T, Kirkland JL. Aging, cell senescence, and chronic disease: emerging therapeutic strategies. JAMA. 2018;320(13):1319‐1320. 30242336 10.1001/jama.2018.12440

[236] Zhang X, Englund DA, Aversa Z, Jachim SK, White TA, LeBrasseur NK. Exercise counters the age-related accumulation of senescent cells. Exerc Sport Sci Rev. 2022;50(4):213‐221. 35776782 10.1249/JES.0000000000000302 PMC9680689

[237] Wiley CD, Campisi J. The metabolic roots of senescence: mechanisms and opportunities for intervention. Nat Metab. 2021;3(10):1290‐1301. 34663974 10.1038/s42255-021-00483-8 PMC8889622

[238] Miller RA, Buehner G, Chang Y, Harper JM, Sigler R, Smith-Wheelock M. Methionine-deficient diet extends mouse lifespan, slows immune and lens aging, alters glucose, T4, IGF-I and insulin levels, and increases hepatocyte MIF levels and stress resistance. Aging Cell. 2005;4(3):119‐125. 15924568 10.1111/j.1474-9726.2005.00152.x PMC7159399

[239] Newman JC, Covarrubias AJ, Zhao M, et al Ketogenic diet reduces midlife mortality and improves memory in aging mice. Cell Metab. 2017;26(3):547‐557.e8. 28877458 10.1016/j.cmet.2017.08.004 PMC5605815

[240] Roberts MN, Wallace MA, Tomilov AA, et al A ketogenic diet extends longevity and healthspan in adult mice. Cell Metab. 2017;26(3):539‐546.e5. 28877457 10.1016/j.cmet.2017.08.005 PMC5609489

[241] Nilsson MI, Bourgeois JM, Nederveen JP, et al Lifelong aerobic exercise protects against inflammaging and cancer. PLoS One. 2019;14(1):e0210863. 30682077 10.1371/journal.pone.0210863 PMC6347267

[242] Garcia-Valles R, Gomez-Cabrera MC, Rodriguez-Mañas L, et al Life-long spontaneous exercise does not prolong lifespan but improves health span in mice. Longev Healthspan. 2013;2(1):14. 24472376 10.1186/2046-2395-2-14 PMC3922914

[243] Schafer MJ, Mazula DL, Brown AK, et al Late-life time-restricted feeding and exercise differentially alter healthspan in obesity. Aging Cell. 2019;18(4):e12966. 31111669 10.1111/acel.12966 PMC6612646

[244] Wen J, Bao M, Tang M, He X, Yao X, Li L. Low magnitude vibration alleviates age-related bone loss by inhibiting cell senescence of osteogenic cells in naturally senescent rats. Aging (Albany NY). 2021;13(8):12031‐12045. 33888646 10.18632/aging.202907 PMC8109117

[245] Simpson RJ, Cosgrove C, Chee MM, et al Senescent phenotypes and telomere lengths of peripheral blood T-cells mobilized by acute exercise in humans. Exerc Immunol Rev. 2010;16:40‐55. 20839490

[246] Walton RG, Kosmac K, Mula J, et al Human skeletal muscle macrophages increase following cycle training and are associated with adaptations that may facilitate growth. Sci Rep. 2019;9(1):969. 30700754 10.1038/s41598-018-37187-1 PMC6353900

[247] Nielsen HB, Secher NH, Christensen NJ, Pedersen BK. Lymphocytes and NK cell activity during repeated bouts of maximal exercise. Am J Physiol. 1996;271(1):R222‐R227. 8760224 10.1152/ajpregu.1996.271.1.R222

[248] Krizhanovsky V, Yon M, Dickins RA, et al Senescence of activated stellate cells limits liver fibrosis. Cell. 2008;134(4):657‐667. 18724938 10.1016/j.cell.2008.06.049 PMC3073300

[249] Shimizu I, Yoshida Y, Katsuno T, et al p53-induced adipose tissue inflammation is critically involved in the development of insulin resistance in heart failure. Cell Metab. 2012;15(1):51‐64. 22225876 10.1016/j.cmet.2011.12.006

[250] Krimpenfort P, Quon KC, Mooi WJ, Loonstra A, Berns A. Loss of p16Ink4a confers susceptibility to metastatic melanoma in mice. Nature. 2001;413(6851):83‐86. 11544530 10.1038/35092584

[251] Martín-Caballero J, Flores JM, García-Palencia P, Serrano M. Tumor susceptibility of p21(Waf1/Cip1)-deficient mice. Cancer Res. 2001;61(16):6234‐6238. 11507077

[252] Sharpless NE, Bardeesy N, Lee KH, et al Loss of p16Ink4a with retention of p19Arf predisposes mice to tumorigenesis. Nature. 2001;413(6851):86‐91. 11544531 10.1038/35092592

[253] Takeuchi S, Takahashi A, Motoi N, et al Intrinsic cooperation between p16INK4a and p21Waf1/Cip1 in the onset of cellular senescence and tumor suppression in vivo. Cancer Res. 2010;70(22):9381‐9390. 21062974 10.1158/0008-5472.CAN-10-0801

[254] Di Micco R, Krizhanovsky V, Baker D, d’Adda di Fagagna F. Cellular senescence in ageing: from mechanisms to therapeutic opportunities. Nat Rev Mol Cell Biol. 2021;22(2):75‐95. 33328614 10.1038/s41580-020-00314-w PMC8344376

[255] Wang B, Wang L, Gasek NS, et al An inducible p21-Cre mouse model to monitor and manipulate p21-highly-expressing senescent cells in vivo. Nat Aging. 2021;1(10):962‐973. 35024619 10.1038/s43587-021-00107-6 PMC8746571

[256] Wang L, Wang B, Gasek NS, et al Targeting p21(Cip1) highly expressing cells in adipose tissue alleviates insulin resistance in obesity. Cell Metab. 2022;34(1):186. 34986334 10.1016/j.cmet.2021.12.014 PMC8832725

[257] Ramos FJ, Chen SC, Garelick MG, et al Rapamycin reverses elevated mTORC1 signaling in lamin A/C-deficient mice, rescues cardiac and skeletal muscle function, and extends survival. Sci Transl Med. 2012;4(144):144ra103. 10.1126/scitranslmed.3003802 PMC3613228 22837538

[258] Daneshgar N, Rabinovitch PS, Dai DF. TOR signaling pathway in cardiac aging and heart failure. Biomolecules. 2021;11(2):168. 33513917 10.3390/biom11020168 PMC7911348

[259] Laberge RM, Sun Y, Orjalo AV, et al MTOR regulates the pro-tumorigenic senescence-associated secretory phenotype by promoting IL1A translation. Nat Cell Biol. 2015;17(8):1049‐1061. 26147250 10.1038/ncb3195 PMC4691706

[260] Spilman P, Podlutskaya N, Hart MJ, et al Inhibition of mTOR by rapamycin abolishes cognitive deficits and reduces amyloid-beta levels in a mouse model of Alzheimer's disease. PLoS One. 2010;5(4):e9979. 20376313 10.1371/journal.pone.0009979 PMC2848616

[261] Hurez V, Dao V, Liu A, et al Chronic mTOR inhibition in mice with rapamycin alters T, B, myeloid, and innate lymphoid cells and gut flora and prolongs life of immune-deficient mice. Aging Cell. 2015;14(6):945‐956. 26315673 10.1111/acel.12380 PMC4693453

[262] Walters HE, Cox LS. mTORC inhibitors as broad-spectrum therapeutics for age-related diseases. Int J Mol Sci. 2018;19(8):2325. 30096787 10.3390/ijms19082325 PMC6121351

[263] Kandhaya-Pillai R, Yang X, Tchkonia T, Martin GM, Kirkland JL, Oshima J. TNF-α/IFN-γ synergy amplifies senescence-associated inflammation and SARS-CoV-2 receptor expression via hyper-activated JAK/STAT1. Aging Cell. 2022;21(6):e13646. 35645319 10.1111/acel.13646 PMC9197409

[264] Xu M, Tchkonia T, Ding H, et al JAK inhibition alleviates the cellular senescence-associated secretory phenotype and frailty in old age. Proc Natl Acad Sci U S A. 2015;112(46):E6301‐E6310. 26578790 10.1073/pnas.1515386112 PMC4655580

[265] Addinsall AB, Cacciani N, Akkad H, et al JAK/STAT inhibition augments soleus muscle function in a rat model of critical illness myopathy via regulation of complement C3/3R. J Physiol. 2021;599(11):2869‐2886. 33745126 10.1113/JP281220

[266] Verstovsek S, Kantarjian H, Mesa RA, et al Safety and efficacy of INCB018424, a JAK1 and JAK2 inhibitor, in myelofibrosis. N Engl J Med. 2010;363(12):1117‐1127. 20843246 10.1056/NEJMoa1002028 PMC5187954

[267] Moiseeva O, Deschênes-Simard X, St-Germain E, et al Metformin inhibits the senescence-associated secretory phenotype by interfering with IKK/NF-κB activation. Aging Cell. 2013;12(3):489‐498. 23521863 10.1111/acel.12075

[268] Effect of intensive blood-glucose control with metformin on complications in overweight patients with type 2 diabetes (UKPDS 34). UK Prospective Diabetes Study (UKPDS) group. Lancet. 1998;352(9131):854‐865. 9742977

[269] Barzilai N, Crandall JP, Kritchevsky SB, Espeland MA. Metformin as a tool to target aging. Cell Metab. 2016;23(6):1060‐1065. 27304507 10.1016/j.cmet.2016.05.011 PMC5943638

[270] Huffman DM, Justice JN, Stout MB, Kirkland JL, Barzilai N, Austad SN. Evaluating health span in preclinical models of aging and disease: guidelines, challenges, and opportunities for geroscience. J Gerontol A Biol Sci Med Sci. 2016;71(11):1395‐1406. 27535967 10.1093/gerona/glw106 PMC5055649

[271] Bannister CA, Holden SE, Jenkins-Jones S, et al Can people with type 2 diabetes live longer than those without? A comparison of mortality in people initiated with metformin or sulphonylurea monotherapy and matched, non-diabetic controls. Diabetes Obes Metab. 2014;16(11):1165‐1173. 25041462 10.1111/dom.12354

[272] American Federation for Aging Research . Targeting the biology of aging. ushering a new era of interventions. Accessed April 5, 2024. https://www.afar.org/tame-trial

[273] Justice JN, Ferrucci L, Newman AB, et al A framework for selection of blood-based biomarkers for geroscience-guided clinical trials: report from the TAME biomarkers workgroup. Geroscience. 2018;40(5-6):419‐436. 30151729 10.1007/s11357-018-0042-y PMC6294728

[274] Quarles E, Basisty N, Chiao YA, et al Rapamycin persistently improves cardiac function in aged, male and female mice, even following cessation of treatment. Aging Cell. 2020;19(2):e13086. 31823466 10.1111/acel.13086 PMC6996961

[275] Baker DJ, Wijshake T, Tchkonia T, et al Clearance of p16Ink4a-positive senescent cells delays ageing-associated disorders. Nature. 2011;479(7372):232‐236. 22048312 10.1038/nature10600 PMC3468323

[276] Pajvani UB, Trujillo ME, Combs TP, et al Fat apoptosis through targeted activation of caspase 8: a new mouse model of inducible and reversible lipoatrophy. Nat Med. 2005;11(7):797‐803. 15965483 10.1038/nm1262

[277] Kirkland JL, Tchkonia T. Senolytic drugs: from discovery to translation. J Intern Med. 2020;288(5):518‐536. 32686219 10.1111/joim.13141 PMC7405395

[278] Wissler Gerdes EO, Zhu Y, Tchkonia T, Kirkland JL. Discovery, development, and future application of senolytics: theories and predictions. FEBS J. 2020;287(12):2418‐2427. 32112672 10.1111/febs.15264 PMC7302972

[279] Mikawa R, Suzuki Y, Baskoro H, et al Elimination of p19(ARF) -expressing cells protects against pulmonary emphysema in mice. Aging Cell. 2018;17(5):e12827. 30058137 10.1111/acel.12827 PMC6156494

[280] Wang E . Senescent human fibroblasts resist programmed cell death, and failure to suppress bcl2 is involved. Cancer Res. 1995;55(11):2284‐2292. 7757977

[281] Passos JF, Saretzki G, Ahmed S, et al Mitochondrial dysfunction accounts for the stochastic heterogeneity in telomere-dependent senescence. PLoS Biol. 2007;5(5):e110. 17472436 10.1371/journal.pbio.0050110 PMC1858712

[282] Wang Y, Chang J, Liu X, et al Discovery of piperlongumine as a potential novel lead for the development of senolytic agents. Aging (Albany NY). 2016;8(11):2915‐2926. 27913811 10.18632/aging.101100 PMC5191878

[283] Kirkland JL, Tchkonia T. Cellular senescence: a translational perspective. EBioMedicine. 2017;21:21‐28. 28416161 10.1016/j.ebiom.2017.04.013 PMC5514381

[284] Passos JF, Nelson G, Wang C, et al Feedback between p21 and reactive oxygen production is necessary for cell senescence. Mol Syst Biol. 2010;6(1):347. 20160708 10.1038/msb.2010.5 PMC2835567

[285] Chang Q, Jorgensen C, Pawson T, Hedley DW. Effects of dasatinib on EphA2 receptor tyrosine kinase activity and downstream signalling in pancreatic cancer. Br J Cancer. 2008;99(7):1074‐1082. 18797457 10.1038/sj.bjc.6604676 PMC2567084

[286] Xi HQ, Wu XS, Wei B, Chen L. Eph receptors and ephrins as targets for cancer therapy. J Cell Mol Med. 2012;16(12):2894‐2909. 22862837 10.1111/j.1582-4934.2012.01612.x PMC4393718

[287] Chang J, Wang Y, Shao L, et al Clearance of senescent cells by ABT263 rejuvenates aged hematopoietic stem cells in mice. Nat Med. 2016;22(1):78‐83. 26657143 10.1038/nm.4010 PMC4762215

[288] Zhu Y, Tchkonia T, Fuhrmann-Stroissnigg H, et al Identification of a novel senolytic agent, navitoclax, targeting the Bcl-2 family of anti-apoptotic factors. Aging Cell. 2016;15(3):428‐435. 26711051 10.1111/acel.12445 PMC4854923

[289] Zhu Y, Doornebal EJ, Pirtskhalava T, et al New agents that target senescent cells: the flavone, fisetin, and the BCL-X(L) inhibitors, A1331852 and A1155463. Aging (Albany NY). 2017;9(3):955‐963. 28273655 10.18632/aging.101202 PMC5391241

[290] Yousefzadeh MJ, Zhu Y, McGowan SJ, et al Fisetin is a senotherapeutic that extends health and lifespan. EBioMedicine. 2018;36:18‐28. 30279143 10.1016/j.ebiom.2018.09.015 PMC6197652

[291] Xu Q, Fu Q, Li Z, et al The flavonoid procyanidin C1 has senotherapeutic activity and increases lifespan in mice. Nat Metab. 2021;3(12):1706‐1726. 34873338 10.1038/s42255-021-00491-8 PMC8688144

[292] Baar MP, Brandt RMC, Putavet DA, et al Targeted apoptosis of senescent cells restores tissue homeostasis in response to chemotoxicity and aging. Cell. 2017;169(1):132‐147.e16. 28340339 10.1016/j.cell.2017.02.031 PMC5556182

[293] Guerrero A, Herranz N, Sun B, et al Cardiac glycosides are broad-spectrum senolytics. Nat Metab. 2019;1(11):1074‐1088. 31799499 10.1038/s42255-019-0122-z PMC6887543

[294] Triana-Martínez F, Picallos-Rabina P, Da Silva-Álvarez S, et al Identification and characterization of Cardiac Glycosides as senolytic compounds. Nat Commun. 2019;10(1):4731. 31636264 10.1038/s41467-019-12888-x PMC6803708

[295] Johmura Y, Yamanaka T, Omori S, et al Senolysis by glutaminolysis inhibition ameliorates various age-associated disorders. Science. 2021;371(6526):265‐270. 33446552 10.1126/science.abb5916

[296] Suda M, Shimizu I, Katsuumi G, et al Glycoprotein nonmetastatic melanoma protein B regulates lysosomal integrity and lifespan of senescent cells. Sci Rep. 2022;12(1):6522. 35444208 10.1038/s41598-022-10522-3 PMC9021310

[297] Yoshida S, Nakagami H, Hayashi H, et al The CD153 vaccine is a senotherapeutic option for preventing the accumulation of senescent T cells in mice. Nat Commun. 2020;11(1):2482. 32424156 10.1038/s41467-020-16347-w PMC7235045

[298] Amor C, Feucht J, Leibold J, et al Senolytic CAR T cells reverse senescence-associated pathologies. Nature. 2020;583(7814):127‐132. 32555459 10.1038/s41586-020-2403-9 PMC7583560

[299] Samakkarnthai P, Saul D, Zhang L, et al In vitro and in vivo effects of zoledronic acid on senescence and senescence-associated secretory phenotype markers. Aging (Albany NY). 2023;15(9):3331‐3355. 37154858 10.18632/aging.204701 PMC10449299

[300] Takahashi K, Yamanaka S. Induction of pluripotent stem cells from mouse embryonic and adult fibroblast cultures by defined factors. Cell. 2006;126(4):663‐676. 16904174 10.1016/j.cell.2006.07.024

[301] Ocampo A, Reddy P, Martinez-Redondo P, et al In vivo amelioration of age-associated hallmarks by partial reprogramming. Cell. 2016;167(7):1719‐1733.e12. 27984723 10.1016/j.cell.2016.11.052 PMC5679279

[302] Xu M, Bradley EW, Weivoda MM, et al Transplanted senescent cells induce an osteoarthritis-like condition in mice. J Gerontol A Biol Sci Med Sci. 2017;72(6):780‐785. 27516624 10.1093/gerona/glw154 PMC5861939

[303] Gonzalez-Meljem JM, Martinez-Barbera JP. Adamantinomatous craniopharyngioma as a model to understand paracrine and senescence-induced tumourigenesis. Cell Mol Life Sci. 2021;78(10):4521‐4544. 34019103 10.1007/s00018-021-03798-7 PMC8195904

[304] Gonzalez-Meljem JM, Haston S, Carreno G, et al Stem cell senescence drives age-attenuated induction of pituitary tumours in mouse models of paediatric craniopharyngioma. Nat Commun. 2017;8(1):1819. 29180744 10.1038/s41467-017-01992-5 PMC5703905

[305] Justice JN, Nambiar AM, Tchkonia T, et al Senolytics in idiopathic pulmonary fibrosis: results from a first-in-human, open-label, pilot study. EBioMedicine. 2019;40:554‐563. 30616998 10.1016/j.ebiom.2018.12.052 PMC6412088

[306] Takasugi M, Yoshida Y, Hara E, Ohtani N. The role of cellular senescence and SASP in tumour microenvironment. FEBS J. 2023;290(5):1348‐1361. 35106956 10.1111/febs.16381

[307] Rao SG, Jackson JG. SASP: tumor suppressor or promoter? Yes!. Trends Cancer. 2016;2(11):676‐687. 28741506 10.1016/j.trecan.2016.10.001

